# Brain Insulin Resistance and Deficiency as Therapeutic Targets in Alzheimer's Disease

**DOI:** 10.2174/156720512799015037

**Published:** 2012-01

**Authors:** Suzanne M de la Monte

**Affiliations:** Departments of Medicine, Pathology, Neurology, and Neurosurgery, Rhode Island Hospital and the Warren Alpert Medical School of Brown University, Providence, RI, USA

**Keywords:** Alzheimer’s disease, dementia, neurofibrillary tangles, neurodegeneration cascade.

## Abstract

Alzheimer's disease [AD] is the most common cause of dementia in North America. Despite 30+ years of intense investigation, the field lacks consensus regarding the etiology and pathogenesis of sporadic AD, and therefore we still do not know the best strategies for treating and preventing this debilitating and costly disease. However, growing evidence supports the concept that AD is fundamentally a metabolic disease with substantial and progressive derangements in brain glucose utilization and responsiveness to insulin and insulin-like growth factor [IGF] stimulation. Moreover, AD is now recognized to be heterogeneous in nature, and not solely the end-product of aberrantly processed, misfolded, and aggregated oligomeric amyloid-beta peptides and hyperphosphorylated tau. Other factors, including impairments in energy metabolism, increased oxidative stress, inflammation, insulin and IGF resistance, and insulin/IGF deficiency in the brain should be incorporated into all equations used to develop diagnostic and therapeutic approaches to AD. Herein, the contributions of impaired insulin and IGF signaling to AD-associated neuronal loss, synaptic disconnection, tau hyperphosphorylation, amyloid-beta accumulation, and impaired energy metabolism are reviewed. In addition, we discuss current therapeutic strategies and suggest additional approaches based on the hypothesis that AD is principally a metabolic disease similar to diabetes mellitus. Ultimately, our ability to effectively detect, monitor, treat, and prevent AD will require more efficient, accurate and integrative diagnostic tools that utilize clinical, neuroimaging, biochemical, and molecular biomarker data. Finally, it is imperative that future therapeutic strategies for AD abandon the concept of uni-modal therapy in favor of multi-modal treatments that target distinct impairments at different levels within the brain insulin/IGF signaling cascades.

## ALZHEIMER'S DISEASE AND BRAIN GLUCOSE METABOLISM

Alzheimer's disease [AD] is the most common cause of dementia in North America, and over the past several decades, the prevalence rates of sporadic AD have become epidemic [1]. Although the clinical diagnosis of AD is based on criteria set by the National Institute of Neurological and Communicative Disorders and Stroke and the Alzheimer's Disease and Related Disorders Association (NINCDS/ ADRDA) and DSM-IV criteria [2], embracement of additional tools such as neuroimaging and standardized biomarker panels could facilitate early detection of disease [3]. Characteristic neuropathological hallmarks of AD include: neuronal loss, abundant accumulations of abnormal, hyperphosphorylated cytoskeletal proteins in neuronal perikarya and dystrophic fibers, and increased expression and abnormal processing of amyloid-beta precursor protein (AβPP), leading to AβPP-Aβ peptide deposition in neurons, plaques, and vessels. For nearly three decades, the dominant trends have been to interpret selected AD-associated abnormalities, namely the hyper-phosphorylation of tau and deposition of AβPP-Aβ as causal rather than consequential to the neurodegeneration cascade. This approach posed significant limitations on the scope of investigation and the goals with respect to designing new treatments; ergo, success has been either modest or disappointing. On the other hand, due to collected contributions of a number of researchers, the field has recently become more receptive to alternative concepts, opening the doors to exciting new avenues of investigation and therapeutic strategies.

Growing evidence supports the concept that AD fundamentally represents a metabolic disease in which brain glucose utilization and energy production are impaired [4-8]. Metabolic abnormalities have been linked to brain insulin and insulin-like growth factor (IGF) resistance with disruption of signaling pathways that regulate neuronal survival, energy production, gene expression, and plasticity [4]. On a cellular basis, inhibition of insulin/IGF signaling contributes to AD-type neurodegeneration by increasing: 1) the activity of kinases that aberrantly phosphorylate tau; 2) expression of AβPP and accumulation of AβPP-Aβ; 3) levels of oxidative and endoplasmic reticulum (ER) stress; 4) the generation of reactive oxygen and reactive nitrogen species that damage proteins, RNA, DNA, and lipids; 5) mitochondrial dysfunction; and 6) activation of pro-inflammatory and pro-death cascades. On a functional basis, insulin/IGF resistance causes down-regulation of target genes that are needed for cholinergic homeostasis, and it compromises systems that mediate neuronal plasticity, memory, and cognition. 

The gold standard for definitively diagnosing AD is to perform a postmortem examination of the brain, with the objective of demonstrating beyond-normal aging associated densities of neurofibrillary tangles, neuritic plaques, and AβPP-Aβ deposits in corticolimbic structures, bearing in mind that neurodegeneration frequently involves multiple other cortical regions as well. The common thread among these characteristic lesions is that they harbor insoluble aggregates of abnormally phosphorylated and ubiquitinated tau, and neurotoxic AβPP-Aβ in the form of oligomers, fibrillar aggregates, or extracellular plaques. Secreted AβPP-Aβ oligomers have been demonstrated to be neurotoxic and to inhibit hippocampal long-term potentiation, i.e. synaptic plasticity [9]. 

Ultimately, to improve our capacity for diagnosis and treatment, we should be able to connect the development and progression of neuropathological lesions with the molecular, biochemical, physiological, neuro-imaging, and clinical abnormalities that correlate with AD. Therefore, gaining a better understanding of the pathophysiology of these lesions could improve our current diagnostic and treatment approaches to AD. One way to begin the process in earnest is to acknowledge that the rigid employment of standardized criteria for diagnosing AD, in fact, restricts our ability to fully comprehend the underlying disease process. For example, in addition to the characteristic lesions noted above, AD is associated with loss of neurons, fibers, and synapses, disruption of the cortical-laminar architecture, gliosis, proliferation of dystrophic neurites, and neuro-inflammatory responses, including microglial cell activation. For unclear reasons, these abnormalities are not systematically quantified, and consequently, they are not routinely incorporated into the AD diagnostic equation. At the same time, many basic cellular, molecular, biochemical, and structural abnormalities in AD overlap with those in other neurodegenerative diseases such as dementia with Lewy bodies, fronto-temporal dementias, and multiple systems atrophy, indicating that one or two biomarkers might not be sufficient to consistently and accurately diagnose AD. 

Hints that AD could represent a metabolic disease emerged from studies showing that the early stages of AD were marked by deficits cerebral glucose utilization [10-15], and that as the disease progressed, metabolic and physiological abnormalities worsened [16, 17]. Subsequently, AD was shown to be associated with brain insulin resistance and insulin deficiency, with significant abnormalities in the expression of genes and activation of kinases that are regulated by insulin and insulin-like growth factor (IGF) signaling [4-8]. Moreover, it was shown that in AD, progressive declines in cerebral glucose utilization, and deficits in insulin signaling and insulin-responsive gene expression worsen with severity of disease. In particular, insulin/IGF regulated genes, including choline acetyltransferase, tau, and glyceraldehyde-3-phosphate dehydrogenase (GAPDH), which mediate cholinergic/cognitive, neuronal cytoskeletal, and metabolic functions, are suppressed in AD [7]. Insulin resistance mediated impairments in energy metabolism lead to oxidative stress, generation of reactive oxygen species (ROS), DNA damage, and mitochondrial dysfunction, all of which drive pro-apoptosis, pro-inflammatory, and pro-AβPP-Aβ cascades. Experimental animals in which brain insulin receptor expression and function were suppressed exhibited cognitive impairment and neurodegeneration with features that overlap with AD [18-22]. 

In AD brains, deficits in insulin/IGF signaling are due to the combined effects of insulin/IGF resistance and deficiency. Insulin/IGF resistance is manifested by reduced levels of insulin/IGF receptor binding and decreased responsiveness to insulin/IGF stimulation, while the trophic factor deficiency is associated with reduced levels of insulin polypeptide and gene expression in brain and cerebrospinal fluid [6-8, 23-25]. In essence, AD can be regarded as a form of brain diabetes that has elements of both insulin resistance and insulin deficiency. To consolidate this concept, we proposed that AD be referred to as, “Type 3 diabetes” [7, 8]. 

## INSULIN AND INSULIN-LIKE GROWTH FACTOR ACTIONS IN THE BRAIN

In the central nervous system (CNS), insulin and IGF signaling play critical roles in regulating and maintaining cognitive function. Insulin, IGF-1 and IGF-2 polypeptide and receptor genes are expressed in neurons [26-28] and glial cells [29-32] throughout the brain, and their highest levels of expression are in structures typically targeted by neurodegenerative diseases [33, 34]. Insulin and IGFs regulate a broad range of neuronal functions throughout life, from embryonic and fetal development to adulthood. The corresponding signaling pathways are activated by insulin and IGF binding to their own receptors, resulting in phosphorylation and activation of intrinsic receptor tyrosine kinases. Subsequent interactions between the phosphorylated receptors and insulin receptor substrate (IRS) molecules promote transmission of downstream signals that inhibit apoptosis, and stimulate growth, survival, metabolism, and plasticity. Anti-apoptotic mechanisms inhibited by insulin/IGF stimulation include BAD (inhibitor of Bcl-2), Forkhead Box O (FoxO), glycogen synthase kinase 3β (GSK-3β), and nuclear factor kappa B (NF-κB). GSK-3β regulates Wnt signaling by phosphorylating β-catenin and thereby targeting it for ubiquitin/proteosome-mediated degradation. Wnt signaling mediates synaptic plasticity in the CNS. Therefore, major functions supported by the insulin/IGF signaling axis include, neuronal growth, survival, differentiation, migration, energy metabolism, gene expression, protein synthesis, cytoskeletal assembly, synapse formation, neurotransmitter function, and plasticity [26, 35-38]. Correspondingly, impaired signaling through insulin and IGF receptors has dire consequences with respect to the structural and functional integrity of the CNS. 

## IMPAIRED INSULIN/IGF SIGNALING AND TAU PATHOLOGY IN AD

The major neuronal cytoskeletal lesions that correlate with severity of dementia in AD, including neurofibrillary tangles and dystrophic neurites, contain aggregated and ubiquitinated insoluble fibrillar tau. In other words, tau accumulation and pathology are the most significant structural correlates of dementia in AD [39, 40]. In AD, tau, a microtubule-associated protein, gets hyperphosphorylated due to inappropriate activation of several proline-directed kinases, including GSK-3β. As a result, tau protein misfolds and self-aggregates into insoluble fibrillar structures [paired helical filaments and straight filaments] that form neurofibrillary tangles, dystrophic neurites, and neuropil threads [41]. Intra-neuronal accumulations of fibrillar tau disrupt neuronal cytoskeletal networks and axonal transport, leading to synaptic disconnection and progressive neurodegeneration [41]. Besides fibrillar tau, pre-fibrillar tau can aggregate, forming soluble tau oligomers or insoluble granular tau, which contribute to neurodegeneration by causing synaptic disconnection and neuronal death [42]. The eventual ubiquitination of hyper-phosphorylated tau [43], combined with dysfunction of the ubiquitin-proteasome system [44], cause further accumulation of insoluble fibrillar tau, oxidative stress, and ROS generation, which together promote neuronal apoptosis, mitochondrial dysfunction, and necrosis in AD [45]. 

**Fig. (1):**
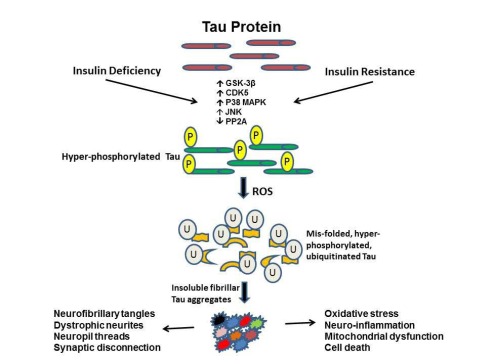
Roles of brain insulin deficiency and brain insulin resistance in Tau pathology. Tau protein is normally regulated by insulin and IGF signalling. Insulin deficiency [effective trophic factor withdrawal] and insulin resistance lead to the over-activation of kinases and inhibition of phosphatases, which result in hyper-phosphorylation of tau. Attendant increased oxidative stress leads to ROS generation and ubiquitination, followed by misfolding of Tau. Misfolded tau aggregates and forms insoluble twisted fibrils that are neurotoxic and mediate dementia-associated neuropathological processes, i.e. neurofibrillary tangle formation, proliferation of dystrophic neuritis and neuropil threads, and synaptic disconnection.

Growing evidence suggests that many of the aforementioned cellular aspects of AD neurodegeneration may be caused by brain insulin/IGF resistance [7, 8] which, as in other brain insulin-resistance states, results in inhibition of downstream pro-growth and pro-survival signaling pathways (Fig. **1**) [46-49]. Tau gene expression and phosphorylation are regulated by insulin and IGF stimulation [50, 51]. In AD, brain insulin and IGF resistance result in decreased signaling through phosphoinositol-3-kinase (PI3K), Akt [50, 51], and Wnt/β-catenin [52], and increased activation of glycogen synthase kinase 3β (GSK-3β) [53-57]. GSK-3β over-activation is partly responsible for the hyper-phosphorylation of *tau,* which leads to tau misfolding and fibril aggregation [58]. In addition, tau hyper-phosphorylation in AD is mediated by increased activation of cyclin-dependent kinase 5 (cdk-5) and c-Abl kinases [59, 60], and inhibition of protein phosphatases 1 and 2A [41, 60, 61]. Besides hyper-phosphorylation, tau pathology in AD is mediated by impaired tau gene expression due to reduced insulin and IGF signaling [62]. Consequences include, failure to generate sufficient quantities of normal soluble tau protein, vis-a-vis accumulation of hyper-phosphorylated insoluble fibillar tau, and attendant exacerbation of cytoskeletal collapse, neurite retraction, and synaptic disconnection. 

## INSULIN/IGF RESISTANCE AND AMYLOID-BETA (AΒ) NEUROTOXICITY

**Fig. (2):**
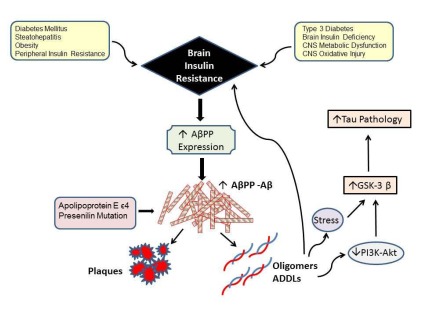
Brain insulin resistance and AβPP-Aβ deposition and toxicity. Brain insulin resistance caused by peripheral insulin resistance diseases or primary toxic and neurodegenerative processes in the brain promote neuroinflammation and increased expression of AβPP. Throught the action of Beta and Gamma secretases, AbPP is cleaved to generate excessive 40-42 kD AβPP-Aβ peptides that aggregate and form insoluble fibrils and plaques, or oligomers and AβPP-Aβ-derived diffusible ligands (ADDLs), which are neurotoxic. AβPP-Aβ oligomers and ADDLs promote oxidative stress and increased activation of kinases that lead to Tau hyperphosphorylation, and its eventual ubiquitination, misfolding, and aggregation. AβPP-Aβ oligomers and ADDLs may also block insulin receptor function and contribute to insulin resistance. Carriers of the ApoE e4 allele or Presenilin mutations are predisposed to excessive and abnormal AβPP cleavage, and AβPP-Aβ accumulation, aggregation, and fibril formation, correlating with increased rates and familial occurrences of AD.

AD is associated with dysregulated expression and processing of amyloid precursor protein (AβPP), resulting in the accumulation of AβPP-Aβ (Aβ) oligomeric fibrils or insoluble larger aggregated fibrils (plaques) that are neurotoxic (Fig. **2**). Pathophysiologically, increased AβPP gene expression, together with altered proteolysis, result in accumulation of 40 or 42 amino acid length Aβ peptides that can aggregate. In familial forms of AD, mutations in the AβPP, presenilin 1 (PS1), and PS2 genes, or inheritance of the Apoliprotein E ε4 (ApoE- ε4) allele, are responsible for increased synthesis and deposition of Aβ peptides in the brain. However, in sporadic AD, which accounts for 90% or more of the cases, the causes of Aβ accumulation and toxicity are still under intense investigation. Over the past few years, interest in the role of impaired insulin/IGF signaling as either the cause or consequence of dysregulated AβPP-Aβ expression and protein processing has grown. 

The concept that Aβ toxicity causes insulin resistance, and the opposing argument that brain insulin resistance with attendant oxidative stress and neuro-inflammation promotes Aβ accumulation and toxicity are both supported by experimental data. For example, studies have established that insulin stimulation accelerates trafficking of Aβ from the trans-Golgi network, where it is generated, to the plasma membrane, and that insulin stimulates Aβ extracellular secretion [63] and inhibits its intracellular accumulation and degradation by insulin-degrading enzyme [64, 65]. Although it remains uncertain as to whether these physiological actions of insulin on AβPP processing contribute to Aβ burden, what is apparent is that impaired insulin signaling can disrupt both the processing of AβPP and clearance of Aβ [66]. The accumulation of Aβ exacerbates the problem because Aβ disrupts insulin signaling by competing with insulin, or reducing the affinity of insulin binding to its own receptor [67, 68]. In addition, AβPP oligomers inhibit neuronal transmission of insulin-stimulated signals by desensitizing and reducing the surface expression of insulin receptors. Furthermore, intracellular AβPP-Aβ directly interferes with PI3 kinase activation of Akt, which leads to impaired survival signaling, increased activation of GSK-3β, and hyper-phosphorylation of tau. Hyper-phosphorylated tau is prone to misfold, aggregate, and become ubiquitinated, leading to the formation of dementia-associated paired-helical filament-containing neuronal cytoskeletal lesions. Since IGF-1 or IGF-2 suppression of GSK-3β activity [69] reduces the neurotoxic effects of AβPP [70-73], the neuro-protective properties of these and related trophic factors could be exploited for therapeutic purposes in AD. 

## INSULIN/IGF RESISTANCE, OXIDATIVE STRESS, AND METABOLIC DYSFUNCTION IN AD

Insulin and IGF signaling pathways regulate glucose utilization, metabolism, and ATP synthesis needed for cellular homeostasis and dynamic modulation of a broad range of functions (Tables **1, 2**). Deficits in cerebral glucose utilization and energy metabolism occur very early in the course of AD, such that they are detectable either prior to, or coincident with the initial stages of cognitive dysfunction [25, 74, 75]. These findings lend strong support the concept that impairments in insulin signaling have important roles in the pathogenesis of AD [8]. Glucose uptake and utilization in brain are dependent upon glucose transport. Glucose transporter 4 (GLUT4) is abundantly expressed along with insulin receptors, in medial temporal lobe structures, which notably are major targets of AD neurodegeneration. Insulin stimulates GLUT4 gene expression and protein trafficking from the cytosol to the plasma membrane to modulate glucose uptake and utilization. Therefore, insulin stimulation of GLUT4 is critical to the regulation of neuronal metabolism and the generation of energy needed for memory and cognition. Although postmortem brain studies have not detected significant reductions in GLUT4 expression in AD [8], the well-documented deficits in brain glucose utilization and energy metabolism vis-a-vis brain insulin/IGF resistance could instead be mediated by impairments in GLUT4 trafficking between the cytosol and plasma membrane.

Deficiencies in energy metabolism tipped by inhibition of insulin/IGF signaling increase oxidative stress, mitochondrial dysfunction, and pro-inflammatory cytokine activation [19, 48, 76]. Oxidative stress leads to increased generation and accumulation of reactive oxygen (ROS) and reactive nitrogen species (RNS) that attack subcellular components and organelles. The resulting chemical modifications, including adducts formed with DNA, RNA, lipids, and proteins, compromise the structural and functional integrity of neurons. Consequences include, loss of cell membrane functions, disruption of the neuronal cytoskeleton with dystrophy and synaptic disconnection, deficits in neurotransmitter function and neuronal plasticity, and perturbation of signal transduction and enzymatic pathways required for energy metabolism, homeostasis, and neuronal survival. 

Mitochondrial dysfunction exacerbates electron transport chain function, reducing ATP generation and increasing ROS production. Pro-inflammatory cytokine activation is mediated by neuro-inflammatory responses in microglia and astrocytes. Neuro-inflammation increases oxidative stress, organelle dysfunction, and pro-apoptosis signaling. Moreover, stresses caused by inhibition of insulin/IGF signaling stimulate AβPP gene expression [77] and aberrant AβPP cleavage, with attendant increased AβPP-Aβ deposition and toxic fibril formation in the brain [73, 78-82]. Persistence of oxidative stress leads to constitutive activation of kinases e.g. GSK-3β, that promote aberrant hyper-phosphorylation of tau. Therefore, in AD, oxidative stress and impairments in energy metabolism stemming from brain insulin/IGF resistance quite likely contribute to neuronal loss, AβPP toxicity, tau cytoskeletal pathology, and neuro-inflammation [7, 26, 83]. The degree to which these abnormalities can be effectively targeted for therapy in AD is actively under investigation.

## MECHANISMS OF BRAIN INSULIN/IGF RESISTANCE IN NEURODEGENERATION [FIG. 3]

Although aging is clearly the dominant risk factor for AD, growing evidence suggests that peripheral insulin resistance with obesity, T2DM, metabolic syndrome (dyslipidemic states), and non-alcoholic steatohepatitis (NASH) mediate brain insulin/IGF resistance, and thereby contribute to the pathogenesis of mild cognitive impairment (MCI), dementia, and AD [5, 6, 25, 26, 50, 51, 84-87]. However, only within the past several years has this field greatly expanded due to input from both human and experimental animal studies that produced new information about the causes and consequences of brain insulin resistance and deficiency in relation to cognitive impairment [7, 8, 22, 83, 88-90]. Concerns over the role of peripheral insulin resistance as a mediator of cognitive impairment and sporadic AD have been ratcheted up by globalization of the obesity epidemic [1, 84]. In order to develop logical and novel approaches for treating and preventing neurodegeneration based on the brain insulin resistance hypothesis, three main questions must be addressed: 1) Do T2DM and other peripheral insulin resistance states cause neurodegeneration, including AD? 2) Do T2DM and other peripheral insulin resistance disease states principally serve as co-factors in the pathogenesis of cognitive impairment and neurodegeneration? or 3) Do T2DM and AD fundamentally represent the same disease processes occurring in different target organs and tissues? These questions are addressed below.

### Contributions of Obesity and T2DM to Cognitive Impairment and Neurodegeneration

Epidemiologic studies demonstrated that individuals with glucose intolerance, deficits in insulin secretion, or T2DM have a significantly increased risk for developing mild cognitive impairment (MCI) or AD-type dementia. Longitudinal studies provided further evidence that T2DM [91, 92] and obesity/dyslipidemic disorders [93] were correlated with later development of MCI, dementia, or AD [91, 94-99]. However, one study showed that obesity itself, with or without superimposed T2DM, increased the risk for MCI, AD, or other forms of neurodegeneration [100], suggesting that systemic factors related to obesity, other than T2DM, can promote neurodegeneration. On the other hand, although a relatively high percentage of individuals with MCI or dementia have T2DM, peripheral insulin resistance, or obesity, the vast majority of patients with AD do not have these diseases. To gain a better understanding of the contributions of T2DM and obesity to neurodegeneration, attention must be given to postmortem human and experimental animal studies.

In general, the arguments made in favor of the concept that T2DM or obesity causes AD are not founded; however, the concept that peripheral insulin resistance disease states contribute to cognitive impairment and AD pathogenesis or progression does have a sound basis. Against a causal role are the findings that, postmortem human brain studies demonstrated no significant increase in AD diagnosis among diabetics [101], and similarly abundant densities of senile plaques and rates of neurofibrillary tangle pathology were observed in subjects with T2DM compared with normal aged controls, although peripheral insulin resistance was more common in AD than with normal aging [102]. Since neurofibrillary tangles and dystrophic neurites are hallmarks of AD and correlate with severity of dementia, the abovementioned findings in human postmortem studies indicate that T2DM alone is not sufficient to cause AD. On the other hand, in experimental mouse and rat models, chronic high fat diet (HFD) feeding and diet induced obesity (DIO) with associated T2DM, do cause cognitive impairment with deficits in spatial learning and memory [103, 104]. Moreover, experimental obesity with T2DM causes mild brain atrophy with brain insulin resistance, neuro-inflammation, oxidative stress, and deficits in cholinergic function [105, 106]. An important qualifier about these studies is that the associated brain abnormalities were typically modest in severity, and they were devoid of the most important structural lesions that characterize AD, i.e. neurofibrillary tangles. Therefore, observations both in humans and experimental models suggest that while obesity or T2DM can be associated with cognitive impairment, mild brain atrophy, and a number of AD-type biochemical and molecular abnormalities in brain, including insulin resistance and oxidative stress, they do not cause significant AD pathology. Instead, the findings suggest that T2DM, obesity, and probably other peripheral/systemic insulin resistance states serve as co-factors contributing to the pathogenesis or progression of neurodegeneration. The significance of these results is that therapeutic strategies designed to treat T2DM, obesity, and systemic insulin resistance could help slow the progress or reduce the severity of AD, but they will not likely prevent it altogether. Correspondingly, a number of studies have already demonstrated that treatment with hypoglycemic or insulin sensitizer agents can be protective in reducing the incidence and severity of AD brain pathology [107].

### Factors Mediating Cognitive Impairment and Neurodegeneration in Disease States with Systemic Insulin Resistance

#### Vascular Factors

The mechanisms by which T2DM, obesity, and peripheral insulin resistance contribute to MCI, dementia, and neurodegeration are not fully understood. Factors under investigation include, chronic hyperglycemia, peripheral insulin resistance, oxidative stress, accumulation of advanced glycation end-products, increased expression and activation of insulin degrading enzyme, increased production of pro-inflammatory cytokines, and cerebral microvascular disease [98]. By far, the contributions of cerebral microvascular disease to AD progression are easiest to sort out, and consequently have been recognized for years. In this regard, postmortem studies demonstrated that similar degrees of dementia occurred in subjects who either had severe AD neuropathology or moderate degrees of AD plus chronic ischemic encephalopathy. The nature of ischemic encephalopathy ranged from multifocal ischemic lesions, to infarcts strategically localized in structures ordinarily targeted for AD neurodegeneration, to leukoaraiosis with extensive attrition of white matter fibers [108]. Magnetic resonance imaging [MRI] studies have lent support to this concept by showing that, among older adults, the risks of developing lacunes and atrophy of medial temporal lobe structures [hippocampal and amygdalar], i.e. targets of AD neurodegeneration, increase with duration and progression of T2DM [109]. Apart from diabetes-associated arteriosclerosis, factors that could contribute to cerebrovascular disease and increased risk of AD progression include, hyperinsulinemia and inheritance of the ApoE-ϵ4 allele. Although controversial, both insulin resistance and hyperinsulinemia are injurious to blood vessels, causing intimal thickening, scarring, and leakiness [110-115]. Furthermore, postmortem brain studies have shown compounded risk for developing AD among hyperinsulinemic diabetics who also carry at least one ApoE-ϵ4 allele, and relative resistance to AD among non-diabetic, ApoE4-ϵ4 negative individuals. The latter group had significantly lower densities of AβPP-Aβ plaques and neurofibrillary tangles compared with ApoE-ϵ4 positive, hyperinsulinemic diabetics.

#### Role of Neurotoxic Lipids Generated in Peripheral Organs and Tissues

Apart from chronic ischemic injury and cerebral microvascular disease, data generated by independent studies suggest that cognitive impairment and neuropsychiatric dysfunction correlate more with hepatic steatosis and insulin resistance than obesity or T2DM [116-122]. For example, neurocognitive deficits and brain insulin resistance occurred in experimental models of chronic HFD feeding in which the mice and rats also developed visceral obesity with hepatic steatosis or steatohepatitis. In addition, toxin exposure models that cause steatohepatitis and hepatic insulin resistance in the absence of obesity, also caused neurodegeneration and cognitive impairment [22, 83, 105, 106, 123, 124]. Therefore, we considered the role of hepatic insulin resistance as a mediator of neurodegeneration. 

Hepatic insulin resistance dysregulates lipid metabolism, resulting in increased oxidative and ER stress, and mitochondrial dysfunction. Mechanistically, physiologic insulin stimulation promotes lipogenesis, which increases triglyceride storage in the liver [125, 126]. However, in disease states associated with injury and inflammation, hepatocytes sustain ER stress, oxidative damage, mitochondrial dysfunction, and lipid peroxidation, which together promote hepatic insulin resistance [125, 127]. Hepatic insulin resistance stimulates lipolysis [128], and lipolysis leads to increased generation of toxic lipids e.g. ceramides, which further impair insulin signaling, mitochondrial function, and cell viability [127, 129, 130]. 

Ceramides are lipid signaling molecules [131] that cause insulin resistance [132-134] by activating pro-inflammatory cytokines [131, 135, 136] and inhibiting signal transduction through PI3 kinase-Akt [137-140]. In diet-induced obesity (DIO) models, insulin resistance develops in adipocytes, along with locally and peripherally increased levels of ceramides [131, 141-145]. With experimental steatohepatitis caused by DIO or low-level nitrosamine exposure, hepatic and peripheral insulin resistance are also accompanied by locally and peripherally increased ceramide levels [22, 83, 105, 123, 124]. The compilation and integration of data from all of these studies led to the realization that cognitive impairment with brain insulin resistance and neurodegeneration was principally correlated with hepatic steatosis-associated insulin resistance rather than obesity or T2DM per se. This point led to the hypothesis that, in the settings of obesity, T2DM, and various peripheral insulin resistance states, cognitive impairment is mediated via a liver-brain axis of neurodegeneration [33, 34, 146].

In essence, the key factors linking peripheral insulin resistance states to cognitive impairment with neurodegeneration and brain insulin resistance, are the increased generation of ceramides in liver, and increased levels of ceramide in peripheral blood. In hepatic dyslipidemic states, ceramide levels increase due to elevated biosynthesis or reduced degradation from altered gene expression and enzymatic activity. *In vitro* experiments showed that ceramides are indeed neurotoxic and cause neuronal insulin resistance, oxidative stress, and molecular and biochemical abnormalities similar to those that occur in AD [147]. Moreover, parenteral administration of cytotoxic ceramides produces sustained impairments in spatial learning and memory with neurodegeneration and brain insulin/IGF resistance, similar to the effects of DIO with T2DM and NASH [146]. Therefore, we hypothesize that toxic lipids, in particular ceramides, generated in livers with hepatic insulin resistance caused by obesity, T2DM, metabolic syndrome, chronic alcohol abuse, or low-dose nitrosamine exposure, are the principal mediators of neurodegeneration, and that they exert their neurotoxic effects in the CNS via a liver-brain axis [146]. Mechanistically, cytotoxic ceramides generated in livers with steatohepatitis, insulin resistance, and ER stress, traffic through the circulation, and due to their lipid soluble nature, they cross the blood-brain barrier and exert neurotoxic and neurodegenerative effects by impairing insulin signaling. Preliminary studies demonstrated that treatment with chemical inhibitors of ceramide biosynthesis enhances insulin sensitivity, and treatment with peroxisome proliferator-activated receptor (PPAR) agonists, which improve insulin responsiveness and reduce oxidative stress [83, 148-150], decrease hepatic ceramide generation, serum ceramide levels, cognitive impairment, and neurodegeneration in models of DIO with T2DM and steatohepatitis [151]. Therefore, in addition to microvascular disease, we propose that peripheral insulin resistance diseases contribute to neurodegeneration, including AD, by increasing production of neurotoxic ceramides that cause brain insulin resistance. This mechanism could account for the parallel epidemics of T2DM, obesity, and AD [1].

### Alzheimer's is a Brain Diabetes Mellitus [Type 3 Diabetes]

A convincing argument could be made that AD, in its pure form, represents a brain form of diabetes mellitus [7, 8] since, AD is associated with progressive brain insulin resistance in the absence of T2DM, obesity, or peripheral insulin resistance [7, 8, 89, 90]. Moreover, postmortem studies demonstrated that the molecular, biochemical, and signal transduction abnormalities in AD are virtually identical to those that occur in T1DM and T2DM [7, 8, 91, 152-156]. The strongest evidence lending support to this concept comes from experimental animal studies in which rats were administered intracerebroventricular injections of streptozotocin, a pro-diabetes drug. The treated rats developed cognitive impairment with deficits in spatial learning and memory, brain insulin resistance and insulin deficiency, and AD-type neurodegeneration, but not diabetes mellitus [22, 157-160]. In contrast, i.p. or i.v. administration of streptozotocin causes diabetes mellitus with relatively mild degrees of hepatic steatosis and neurodegeneration [157, 161-163]. These findings indicate that exposure to a single pro-diabetes drug can cause organ/tissue degeneration characterized by impairments in insulin signaling and energy metabolism, with attendant increased oxidative stress, mitochondrial dysfunction, and cell death. However, disease spectrum and target organ involvement are governed by dose and route of drug administration.

Despite overwhelmingly convincing data, relevance of this specific model to the human condition remains open to question because streptozotocin is generally not available to humans. On the other hand, this hypothesis is rendered more appealing by the realization that streptozotocin is a nitrosamine-related compound, and that over the past several decades, Western societies have been assaulted by continuous and increasing exposures to environmental and food-related nitrosamines. We conducted experiments to determine if low, sub-mutagenic doses of nitrosamine compounds that are found in food, e.g. N-nitrosodiethylamine (NDEA), could cause insulin resistance diseases. Alarmingly, those studies showed that low-dose and very limited exposures to NDEA cause T2DM, non-alcoholic steatohepatitis, visceral obesity, cognitive impairment, and AD-type neurodegeneration with peripheral, hepatic, and brain insulin resistance [123, 124], similar to the effects of streptozotocin. Moreover, the adverse effects of NDEA on neuro-cognitive deficits, peripheral, hepatic, and brain insulin resistance, steatohepatitis, and neurodegeneration were exacerbated by chronic high fat diet feeding [164, 165]. Therefore, depending on the structure of the compound, dose, and route of administration, exposures to nitrosamine-related chemicals can cause insulin resistance diseases in multiple different target organs, including brain. In addition to providing evidence that the relatively recent epidemics of sporadic AD, T2DM, and non-alcoholic steatohepatitis/metabolic syndrome could be mediated by environmental or dietary exposures [1], these studies demonstrate that insulin resistance diseases with essentially the same underlying cellular abnormalities, can develop in various organs and tissues. This phenomenon could account for the overlapping increases in prevalence rates of various insulin resistance diseases within the past several decades, as well as the very frequent but incomplete overlap between AD and obesity, T2DM, and NASH [102], which did not exist prior to 1980, and is not accounted for by aging of the population [1].

### Strategies for Early Diagnosis and Evaluation of Treatment Responses -Neuroimaging

The combined use of clinical and postmortem assessments provides the most accurate means of diagnosing AD. However, further advancements in detection, monitoring and treatment, particularly in the early stages of disease, will not likely occur without additional objective and standardized tools [166]. Although magnetic resonance imaging [MRI] of the brain can be used to track progression of medial temporal lobe atrophy as mild cognitive impairment (MCI) advances toward AD, and AD worsens in stage and severity [167], the diagnostic specificity of such a single-pronged approach is limited [168]. On the other hand, emerging evidence suggests that assessments of brain function, including flow and metabolism, combined with structural abnormalities may substantially improve diagnostic accuracy and help monitor disease progression. For example, the progressive cortical hypo-perfusion and hypo-metabolism that accompany advancement of AD, can be readily detected by single photon emission computed tomography (SPECT]) [169, 170] and positron-emission tomography (PET) [12, 168, 171-174]. Furthermore, tracking deficits in flow and metabolism to specific brain regions that are targeted by AD could help improve diagnostic accuracy. In this regard, detecting metabolic and blood flow impairments in the posterior cingulate and parietal-temporal cortices would support an AD diagnosis [175]. On the other hand, it is unlikely that neuroimaging will ever stand alone as a diagnostic tool since this approach cannot replace sophisticated clinical assessments that detect subtle neurobehavioral abnormalities that help distinguish one form of neurodegeneration from another. In addition, the fact that several types neurodegenerative disease, including ischemic encephalopathy, can overlap with the structural targets in AD, supports the argument that, right now, we need better, non-invasive diagnostic measures that go beyond, but also compliment neuroimaging assessments of brain structure, flow and metabolism. 

Functional MRI adds an important diagnostic dimension because it combines neuro-imaging with assessments of brain metabolic responses to stimuli, including insulin [171, 176]. In addition, the joint use of PET with diffusion tensor imaging to correlate severity of cortical hypo-metabolism with loss of white matter integrity [177] holds promise in terms of improving our ability to diagnose and monitor progression of AD, based on objective stage-dependent structural, functional, and metabolic criteria for neurodegeneration. Detection of leukoaraiosis and white matter high intensity lesions in diabetics could help predict individuals at risk for cognitive impairment and dementia [178]. The expectation is that ultimately, the use of sensitive functional neuroimaging, combined with structural imaging and multimodal biomarker panels, will improve diagnostic accuracy and facilitate evaluation of treatment effects in the early stages of AD [179-181]. 

### Strategies for Early Diagnosis and Evaluation of Treatment Responses - Cerebrospinal Fluid and Peripheral Blood Biomarkers

Biomarkers for detecting and diagnosing severity of AD have largely been focused on measuring AβPP-Aβ, tau, and phospho-tau in cerebrospinal fluid (CSF) [182, 183]. Changes in CSF levels of Aβ-42, total tau, and phospho-tau [181] can help predict progression from MCI to dementia [184], or aid in establishing a diagnosis of AD [185]. At least in some studies, the sensitivity and specificity of these CSF biomarkers approach 85% for diagnosing AD and distinguishing AD from MCI [175, 179, 183, 186-188]. However, inter-laboratory variability and the lack of standardization measures to ensure quality assurance, have limited broad and independent use of these assays [189, 190]. Moreover, since at the core of the neurodegenerative process is protein misfolding and aggregation, biomarkers are needed to identify and quantify oligomeric neurotoxic aggregates of tau and Aβ-42 [191]. Beyond these issues lay concerns that by limiting our considerations to basically two biomarkers, we have failed to significantly advance the field. In fact, for various reasons, this rather circumscribed approach has not proven to be sufficiently sensitive to render an accurate diagnosis of AD or predict outcomes of MCI in a case-by-case basis [192].

Given the spectrum of other significant abnormalities that precede or accompany AD, it would seem more prudent to incorporate a broader spectrum of biomarkers into a multimodal panel to better characterize AD stage and progression [193]. For example, indices of oxidative stress, neuro-inflammation, mitochondrial dysfunction, metabolic derangements, and impaired insulin/IGF signaling should be integrated into the overall equation to improve the sensitivity and specificity of diagnosing AD [3, 193, 194]. The use of multi-analyte profiling would enable efficient capture of data and tracking of abnormalities as the biomarker indices shift with disease progression [195]. For example, CSF pro-inflammatory cytokine levels are elevated in the early stages of AD, as well as in MCI [196]. Similarly, MCI and early-stage AD are marked by increased oxidative stress with raised levels of redox-active iron in CSF [197]. These findings suggest that neuro-inflammatory and oxidative stress responses should be evaluated to help gauge the presence, severity, and progression of neurodegeneration in the early stages of disease. At the same time, it could be argued that these factors may be initially responsible for propagating the neurodegeneration cascade, and therefore should be considered as potential therapeutic targets. Although, in later stages of disease, oxidative stress and pro-inflammatory biomarkers, whether in plasma or CSF, seem to lack diagnostic utility [198], the persistently elevated CSF levels of oxidized coenzyme Q-10 and 8-hydroxy-2'-deoxyguaniosine suggest that mitochondrial and DNA oxidative damage mediate AD progression [199, 200], and therefore could be targeted therapeutically to slow the advancement of AD.

Peripheral blood biomarkers in lymphocytes and plasma hold some promise as non-invasive surrogate screening tools, and may provide a means to study populations at increased risk for developing AD [201]. For example, abnormalities in AβPP-Aβ cleavage are detectable in peripheral blood lymphocytes in AD. In addition, protein kinase C (PKC), which has an important role in stimulating AβPP-Aβ peptide formation and tau hyperphosphorylation, could serve as a peripheral blood biomarker, since conformational changes in the PKC enzyme that promote AD pathology are detectable in erythrocytes [202]. Similarly, from the perspective that neuro-inflammation promotes neurodegeneration, it may be possible to use elevated serum levels of acute phase proteins and pro-inflammatory cytokines to help gauge the likelihood of progressing from MCI to dementia, particularly in the early stages of disease when neuro-inflammation is likely to be a relevant biomarker [203]. However, the utility of peripheral blood cytokine and trophic factor levels as diagnostic aids for distinguishing AD from MCI has proven unacceptable due to disease heterogeneity and the multiple co-factors contributing to neurodegeneration [204]. The combined use of serum and CSF to measure AβPP-Aβ peptides, total tau, and phosphorylated tau has been proposed for diagnosis and monitoring responses to treatment [205]. But, this approach could be flawed because drug treatments may not produce detectable shifts in serum levels of AβPP-Aβ or tau [206]. Again, these limitations highlight the importance of establishing multi-pronged diagnostic approaches that will include CSF and serum bio-assays, together with functional and structural neuro-imaging studies to diagnose AD and predict progression from MCI to AD [183].

In designing comprehensive CNS neurodegenerative disease biomarker panels, it should also be possible to capitalize on the concept that AD is a metabolic disease that closely resembles a brain form of diabetes mellitus. CSF assays could be used to detect brain insulin resistance and insulin deficiency, while peripheral blood studies could be used to simultaneously assess peripheral insulin resistance status marked by reduced glucose tolerance, hyperglycemia, hyperinsulinemia, accumulation of advanced glycation endproducts, and reactive oxygen species [178]. For example, AD is associated with significantly reduced CSF insulin levels, but in some cases, hyper-insulinemia as well [178, 207]. The finding that plasma and CSF insulin levels may be significantly elevated in AD relative to normal aged controls after an oral glucose load, but not at basal or post-fasting time points [11] suggests that insulin resistance in AD should be assessed with dynamic functional rather than static assays. This concept is reinforced by the fact that the extent of these abnormalities correlates with severity of dementia, particularly among individuals who lack the ApoE-ϵ4 allele [208]. On the other hand, not all studies have been able to confirm reduced CSF insulin levels in AD [209]. Finally, in AD, CSF levels of IGF binding proteins [IGFBP] 2 and 6 [210], and both CSF and serum levels of IGF-1 [211] are elevated relative to control [210]. This suggests that, in addition to insulin resistance, AD is associated with IGF-1 resistance in the brain, and therefore attending to just one or the other signaling pathway will likely not be sufficient to arrest disease. 

Besides insulin and IGFs, previous studies have suggested roles for impaired expression and function of other CNS trophic factors in the context of neurodegeneration. For example, the role of nerve growth factor [NGF] as a potential target for both diagnosis and treatment arose because NGF promotes survival and function of basal forebrain cholinergic neurons which undergo neurodegeneration early in the course of AD [212, 213]. However, interest in this concept initially waned, in part due to the finding that CSF and/or brain levels of NGF were not abnormal in AD [214-216]. Although subsequent studies utilizing more sensitive approaches, were able to detect significantly elevated levels of NGF in AD CSF or ventricular fluid [217, 218], similar abnormalities were observed in vascular dementia as well [219]. 

Other trophic factors of note include, transforming growth factor beta (TGFbeta) modulates responses to injury in the brain, acidic fibroblast growth factor (aFGF), which modulates cellular proliferation and differentiation, and neuronal thread protein (NTP), which accumulates in AD brains, is regulated by insulin, and physically interacts with phospho-tau [62, 220-222]. With regard to TGFbeta, CSF studies have produced mixed results with some reports showing no change [223, 224], and others demonstrating significantly elevated levels of TGFbeta [225, 226] in AD relative to controls. In two independent studies, elevated choroid plexus and CSF levels of aFGF distinguished AD from normal aging [227, 228]. Finally, a number of studies published in the last 10-15 years demonstrated the utility of neuronal thread protein (NTP) as a potential CSF biomarker of AD [229-234]. However, the lack of widely available reagents and poor understanding of its connection to AD, prevented its incorporation into mainstream concepts about neurodegeneration. Nonetheless, the aggregate findings with regard to AD-associated proteins other than phospho-tau and AβPP-Aβ, suggest that assays of NGF, TGFbeta, aFGF, and possibly NTP in CSF or ventricular fluid could be employed in multi-biomarker panels for detecting AD or monitoring responses to therapy. 

Ideally, it would be most convenient and least costly to utilize peripheral blood or urine based biomarker assays to detect neurodegeneration. Such minimally invasive approaches have been considered and are still under scrutiny. For example, elevated levels of NTP are detectable in urine of AD patients, beginning early in the course of disease [230, 235-238]. Serum-based immunoassays have been used to detect oxidative injury pertinent to AβPP-Aβ and phospho-tau accumulations [198] or insulin/IGF-related metabolic impairments [210, 211] in AD brains. Paralleling observations with respect to CSF, investigators detected elevated levels of aFGF in AD sera [227]. However, attempts to extend these types of analyses to all potential biomarkers may not be feasible without substantial technical improvements in the sensitivity and specificity of detection methods. For example, even with regard to established abnormalities in AD, e.g. GLP-1, neuropeptide Y, and ghrelin-growth hormone expression, the levels detected in serum do not reliably distinguish patients with neurodegeneration from those with normal aging [239]. Another potential hurdle in designing serum-, plasma- or urine-based assays of AD is that certain neurodegeneration-associated abnormalities, even those that can be detected in CSF, may not be detectable in extra-CNS body fluids. Future studies should be directed toward correlating AD biomarker assay data from CSF, peripheral blood, and urine to establish non-invasive means of detecting and monitoring AD neurodegeneration and responses to therapy.

### Rectifying Dementia Based on the Brain Insulin Resistance/Insulin Deficiency Disease Model to Prevent, Retard, Halt, and Cure AD-Type Neurodegeneration

The volume of literature supporting the concept that AD is associated with deficits in energy metabolism, glucose utilization, and insulin/IGF responsiveness in the brain has grown rapidly, causing the paradigm of AD pathogenesis to shift away from the overwhelmingly dominant amyloid and taupathy hypotheses. The attractiveness of the metabolic/brain insulin resistance hypothesis is that the impairments in brain insulin and IGF signaling caused by insulin/IGF resistance, together with the eventual depletion of trophic factors, could account for nearly all other abnormalities that occur in AD, including increased oxidative stress and ROS generation, mitochondrial dysfunction, cell death, loss of synaptic plasticity, deficits in cholinergic homeostasis, increase expression of AβPP, hyper-phosphorylation of tau, compromised myelin maintenance, and neuroinflammation. Another attractive feature of the metabolic/brain insulin resistance hypothesis is that it demystifies the pathophysiology of AD by relating it to other well-recognized systemic diseases, i.e. diabetes mellitus, non-alcoholic steatohepatitis, and metabolic syndrome. If indeed these diseases are all essentially the same except they involve different principal target organs, then the treatment and prevention approaches would also be similar or possibly the same. This concept is not far-fetched. For example, atherosclerosis can preferentially affect different major blood vessels and result in different patterns and distributions of tissue injury and disease, yet no one would regard each spectrum of disease as having a distinct pathogenesis. However, what does require further research is determining the degrees to which brain insulin resistance, cognitive impairment, and neurodegeneration are consequential to peripheral insulin resistance diseases, particularly type 2 diabetes mellitus (T2DM), obesity, and metabolic syndrome, or whether they constitute an intrinsic disease process equivalent to a brain form of diabetes mellitus. Although the above discussions culling human and experimental animal studies lend support to both arguments, epidemiological, clinical, and postmortem studies clearly show that sporadic AD develops primarily in the absence of obesity and T2DM. Nonetheless, with strong data coming from both points of view, it is likely that both concepts are correct. In fact, the existence of primary and secondary mechanisms of brain insulin resistance and neurodegeneration would help explain the heterogeneity of the AD phenotype. In the ensuing discussion of potential therapeutic targets for AD, emphasis will be placed on how current strategies address the brain insulin resistance/metabolic impairments in AD.

### Potential Therapeutic Targets and Strategies for AD

Regarding potential strategies for treating sporadic AD, the major issues to be considered are: 1) Does hyper-insulinemia in obesity and T2DM cause oxidative stress and neurodegeneration; 2) does peripheral insulin resistance lead to increased cerebral micro-vascular disease and thereby cause brain atrophy and degeneration? 3) How do dyslipidemic states associated with T2DM, obesity, and non-alcoholic steatohepatitis (NASH) contribute to neurodegeneration? 4) does the overlap among T2DM, NASH, obesity, and neurodegeneration/AD reflect a single pathophysiological process that causes variable degrees of metabolic dysregulation in different target organs and tissues, e.g. brain, liver, and skeletal muscle? 5) To what extent is AD-type neurodegeneration primarily mediated by intrinsic impairments of brain insulin/IGF signaling, versus secondary effects of peripheral organ insulin resistance diseases, e.g. T2DM? In many respects, the appreciation that AD fundamentally represents a metabolic disease associated with the same molecular, biochemical, and cell signaling abnormalities identified in peripheral insulin resistance diseases, could simplify future approaches to treatment and prevention (Table **3**). For example, pharmacotherapeutic concepts developed for T2DM and NASH may be adaptable to AD. Already, this concept has been tested, and under some conditional circumstances, positive responses to treatment with intranasal insulin and insulin sensitizer drugs have been observed in subjects with AD [66, 240-246]. However, in order to make real progress with respect to treatment, we must acknowledge that AD is the end result of a neurodegeneration cascade that progressively targets and cripples different aspects of cellular physiology and homeostasis. Therefore, one should anticipate that while mono-therapies may be appropriate early in the course of disease, over time, this approach will be doomed to failure [247]. Such lessons have already been learned in the field oncology. Unless we can completely prevent AD, multi-pronged approaches will be needed to support a range of cellular functions and minimize cellular injury and toxicity as the disease progresses. The rationale and targets of current and potential future therapies for AD are discussed below.

### Cholinergic Deficits and Glutamatergic Dysfunction as Targets

Acetylcholinesterase inhibitors are used to treat AD because acetylcholine levels are significantly reduced in the early stages of AD [75, 248], and the cholinergic system mediates cognitive function and neuronal plasticity. One of the major factors contributing to the acetylcholine deficits in AD is the loss of cholinergic neurons in the basal forebrain [213], as these neurons project widely to the cerebral cortex. A second factor is that choline acetyltransferase expression and activity are reduced even in viable neurons in brains with AD [7, 8]. It is noteworthy that choline acetyltransferase expression and activity are regulated by insulin/IGF stimulation [7, 249]. Although there is no evidence that acetylcholinesterase activity is significantly increased in AD brains, the rationale for treating patients with cholinesterase inhibitors is that such drugs could help sustain normal levels of acetylcholine by reducing the rates of its degradation. In addition, cholinesterase inhibitors may have off-target therapeutic effects by stimulating production of trophic factors, such as growth hormone and IGF-1 [250], which also support cognitive function. A third reason for administering acetylcholinesterase inhibitor drugs is that they may be protective against glutamate neurotoxicity and promote neuronal survival through activation of PI3 kinase-Akt [251]. Currently, the standard of care for AD includes the use of acetylcholinesterase inhibitors such as donepezil, aricept, tacrine, and rivastigmine [252]. 

The more recent addition of glutamate receptor antagonists to the AD treatment regimen is based on the concepts that L-glutamate functions as an excitatory amino acid neurotransmitter, and that glutamatergic dysfunction in AD leads to sustained excitotoxicity with attendant impairment of synaptic plasticity needed for learning and memory [253, 254]. Consequently, antagonists to the N-methyl-D-aspartate (NMDA) glutamatergic receptor, such as memantine (Nemenda), are used in conjunction with cholinesterase inhibitors to support memory in patients with mild to moderate AD [255, 256]. Although these approaches provide moderate symptomatic relief and delay disease progression in the early stages of AD, long-term therapeutic responses are limited at best, as these drugs do not arrest or reverse neurodegeneration [257-260]. Therefore, additional approaches are needed, and should include drugs that target various components of the neurodegeneration cascade. For example, there is evidence suggesting that the combined administration of a acetylcholinesterase inhibitors and anti-oxidants (Formula F or Ginkgo biloba-EGb 761) can more effectively improve cognitive performance than either compound alone [261, 262]. Since NMDA can promote insulin resistance [263], and NMDA neurotoxicity can be attenuated or prevented by insulin stimulation [264]. Therefore, therapeutic measures to inhibit glutamate excitotoxicity may help restore brain metabolic functions, particularly in the context of insulin or insulin sensitizer treatments.

### AβPP-Aβ Accumulation and Production as Therapeutic Targets

Research is extensively focused on finding safe and effective means of depleting the brain of toxic AβPP-Aβ deposits, reducing AβPP-Aβ fibrillarization and aggregation, and preventing abnormal cleavage and processing of AβPP [265]. The overarching hypothesis is that AβPP-Aβ peptides are neurotoxic, promote amyloid plaque formation, and mediate tau hyper-phosphorylation, fibrillarization, and neurofibrillary tangle formation [266]. Efforts to deplete the brain of toxic AβPP-Aβ led to the development of AβPP-Aβ-targeted immunotherapy. Although AβPP-Aβ active immunization with AβPP-Aβ peptides, or passive delivery of AβPP-Aβ-specific antibodies can effectively clear AβPP-Aβ plaques from brains of humans and experimental animals [267], the net outcomes are not very encouraging because the AβPP-Aβ instead accumulates in vessels, increasing propensity for micro-hemorrhage [268]. Even more disappointing were the findings that the effects of AβPP-Aβ clearance on cognitive function have been either modest or undetectable, and that patients receiving the vaccine still died with end-stage dementia and extensive neurofibrillary tangle and neuritic pathology in their brains [269, 270]. Moreover, amyloid vaccination produced unacceptable complications such as encephalitis mediated by auto-reactive T cell responses and vasogenic cerebral edema [267, 270].

Besides the neurological complications of brain swelling, particularly in subjects who did not have significant global brain atrophy, the root causes of vasogenic edema, i.e. pro-inflammatory responses with increased microglial activation, cerebral amyloid angiopathy, and accumulation of soluble neurotoxic oligomeric AβPP-Aβ [271], may have adversely influenced the clinical course of AD. Furthermore, studies examining therapeutic effects of passive humanized AβPP-Aβ antibody immunization demonstrated that despite AβPP-Aβ clearance and ample delivery of the antibodies to the brain [272], significant therapeutic responses such as improved survival or retarded progression from mild or moderate to severe dementia could not be demonstrated; consequently, those clinical trials were halted [273]. Altogether, the results of the AβPP-Aβ immunization trials suggest that, despite promising results in experimental animal models, this approach alone will not likely succeed for treating AD in humans. However, before this chapter can be closed, additional studies are needed to determine the degree to which pharmacological clearance of toxic AβPP-Aβ or preventing its formation and accumulation in brain can preserve or restore cognitive function and slow progression of AD neurodegeneration.

One potential approach to preventing the formation and build-up of toxic AβPP-Aβ is to inhibit the expression or activity of enzymes responsible for aberrant processing and cleavage of AβPP. AβPP-Aβ is generated by sequential proteolysis, first with beta secretases, then gamma-secretases [274]. Presenilins, which are often mutated in early onset familial AD, form the catalytic component of gamma-secretases, which mediate intramembranous cleavage of type 1 transmembrane proteins, including AβPP [275]. Mutation of presenilin genes leads to accumulation of AβPP-Aβ in AD, as well as other aging-associated neurodegenerative diseases, including fronto-temporal lobe and Lewy body dementias [275]. To inhibit abnormal processing of AβPP and the attendant accumulation of toxic AβPP-Aβ, efforts have been focused on targeting gamma secretases, including for the treatment of sporadic, late-onset AD [276, 277]. Although the pharmacological effects of gamma secretase inhibitors proved promising with regard to their ability to effectively lower plasma, CSF, and brain AβPP-Aβ burden [274, 278], the disappointing result was that objective clinical therapeutic responses to these agents proved to be minimal or undetectable [276, 279, 280]. Worse yet, the compounds proved to be highly toxic due to concurrent inhibition of Notch signaling pathways [274, 277]. In the adult brain, Notch signaling mediates neuronal plasticity, cognition, and long-term memory [281].

To circumvent toxicity-related problems, efforts are underway to develop Notch cleavage-sparing gamma secretase inhibitor drugs [282, 283]. Results of clinical trials that may be underway are not yet known. However, besides toxicity, the potential therapeutic effectiveness of gamma secretase inhibitors has been drawn into question because the net effects of these agents on AβPP-Aβ levels may be too low to improve clinical outcome [277]. The same problem exists regarding the use of non-steroidal anti-inflammatory drugs (NSAIDs), which have Notch-sparing gamma secretase inhibitory effects, but they produce weak clinical therapeutic responses [277, 284]. Nonetheless, the potential still exists to develop drugs that reduce AβPP-Aβ burden and toxicity by other mechanisms such as, inhibiting beta site AβPP cleaving enzyme 1 (BACE1), increasing expression of alpha secretase, or blocking AβPP-Aβ peptide fibrillization [252]. 

With regard to insulin resistance and the potential contributions of Aβ toxicity, since insulin accelerates trafficking of Aβ from the trans-Golgi network to the plasma membrane, and extracellular secretion of Aβ [63], and impaired insulin signaling disrupts the processing of AβPP and clearance of Aβ [66], it seems likely that by addressing the underlying causes of insulin/IGF resistance, we may be able to effectively and safely reduce AβPP-Aβ burden in the brain. This point is reinforced by the finding that IGF-1 and IGF-2 are neuroprotective as they reduce the neurotoxic effects of AβPP [70-73]. On the other hand, the fact that AβPP oligomers inhibit neuronal insulin-stimulated signals, blocking PI3 kinase activation of Akt, which leads to impaired survival signaling, increased activation of GSK-3β, and resultant hyper-phosphorylation of tau, argues in favor of pursuing measures that reduce AβPP oligomer fibrillarization as a means of restoring brain insulin sensitivity. 

### Therapeutic Targeting of Tau Hyper-Phosphorylation

Hyperphosphorylation of tau leads to misfolding and aggregation of oligomeric fibrils, followed by ubiquitination and generation of dementia-associate paired helical filaments. Paired-helical filaments form the cores of neurofibrillary tangles, neuropil threads, and dystrophic neurites, which are structural hallmarks of AD neuropathology. Tau hyperphosphorylation is mediated by inappropriate and sustained activation of kinases, including GSK-3β [285], cyclin-dependent kinase -5 (cdk-5), p38 MAPK, and c-jun kinase (JNK) [286, 287], and inhibition of phosphatases that mediate physiological dephosphorylation of tau, e.g. protein phosphatase-2A [287]. Therefore, treatment with chemical inhibitors of one or more of these disease- relevant kinases, or phosphatase activators, may reduce the rates of neurofibrillary pathology in AD. Among these potential targets, somewhat greater attention has been paid to the role of GSK-3β because, in addition to promoting tau hyper-phosphorylation, high levels of GSK-3β activity lead to alterations in AβPP processing and increased neuronal death [285, 288-290]. 

Approaches to therapeutically inhibiting GSK-3β activity have mainly included the use of lithium chloride, and to a lesser extent, indigoids [285, 288-291]. Experimentally, lithium chloride treatment of Swedish AβPP transgenic mice reduced GSK-3β-induced AβPP-Aβ accumulation [292, 293], and treatment of mutant AβPP and tau double transgenic AD mice with the NP12 GSK-3 inhibitor, significantly reduced brain amyloid burden, tau alterations, and neuronal survival [294]. In contrast, lithium treatment of aged 3xTg-AD mice reduced tau phosphorylation, but did not alter AβPP-Aβ load or improve performance on working memory tasks [295]. Therefore, in several well-characterized experimental models, therapeutic inhibition of GSK-3β prevented certain aspects of AD-type neurodegeneration, although the results varied, depending on the underlying gene abnormalities. 

In several uncontrolled or retrospective human clinical studies, it was demonstrated that prior use of lithium therapy in psychiatric patients was protective against dementia and associated with better performance on cognitive tests [296-299]. In addition, among individuals at risk for early onset familial AD, chronic lithium treatment reduced the prevalence rates of AD and the brain activity levels of GSK-3β, and it increased the levels of brain-derived neurotrophic factor (BDNF) [300]. However, a subsequent randomized, single-blind, short-term (10 weeks) placebo-controlled multicenter trial proved disappointing in that performance on standardized cognitive function tests was not significantly improved, and no significant reductions in CSF GSK-3β activity were detected [301]. However, those findings ought to be interpreted with caution, given the relatively short duration of the trial compared with earlier (uncontrolled) retrospective studies.

Altogether, the effects of GSK-3 inhibitor treatments on neurodegeneration and cognitive performance in both human and experimental animal studies have been mixed. Variability in the diminution of GSK-3β-induced pathology, i.e. tau phosphorylation, AβPP-Aβ load, and cognitive function, could be attributed to the inadequacy of controls, differences in the durations of treatment and observation, nature of the underlying mechanisms of neurodegeneration, and variability in outcome measurements. Therefore, it may be premature to abandon this approach without further systematic investigation, particularly since the scientific basis for the overarching hypothesis is sound. Nonetheless, an important caveat with regard to going forward with this therapeutic strategy is that efforts should be made to target only the GSK-3β activity that is aberrantly increased in the CNS, rather than broadly inhibit GSK-3β throughout the brain and body. Since GSK-3β is a broadly acting kinase that regulates signaling through critical pathways such as Wnt and Notch [302, 303], which in the adult CNS are crucial for neural stem cell homeostasis [304, 305], chronic global inhibition of GSK-3β could have unintended consequences with regard to regenerative and repair mechanisms in brain cells that are not targeted for neurodegeneration. 

### Insulin Therapy 

The proposed used of anti-diabetes, hypoglycemic drugs to treat AD is based on the findings that: 1) AD is associated with brain insulin resistance and insulin deficiency (reduced brain and CSF levels), with or without associated systemic insulin resistance or T2DM; 2) diabetic patients that are well-managed with insulin or hypoglycemic medications exhibit significant improvements in memory and slowing of AD progression; 3) treated elderly diabetics have lower densities of AD lesions compared with non-diabetic controls; 4) insulin administration improves cognition and memory in AD, and insulin stimulated cognition is correlated with increased levels of norepinephrine in both plasma and CSF [306]; 5) hyper-insulinemic euglycemic clamping enhances cognition and attention in patients with AD; and 6] experimental intracerebral or intravenous treatments with insulin improve memory, cognition, evoked brain potentials, and neurotransmitter function [66]. Although attractive and seemingly simple, a foremost consideration is the fact that the target population consists of elderly individuals who probably have other chronic diseases and who are at increased risk for problems related to inadvertent bouts of hypoglycemia, e.g. traumatic falls that could be debilitating or life-threatening, and metabolic insults to various organs, including brain. Moreover, the effectiveness of insulin therapy may be dependent upon simultaneously increased levels/availability of glucose, and it may not be effective in facilitating memory if CSF AβPP-Aβ42 levels are markedly elevated due to insulin resistance [307]. Therefore, the routine use of systemic insulin therapy for patients with AD would be impractical and probably ill-advised, if not unacceptable. 

However, another approach taken that avoids potentially harmful side-effects is to administer intranasal insulin. Intranasal insulin increases brain insulin levels and improves performance on declarative memory tasks while having little effect on plasma glucose and insulin levels [308]. In addition, intranasal insulin delivered via an electronic atomizer, improves attention and increases the AβPP-Aβ 40/AβPP-Aβ42 ratio [245]. Reducing the relative amounts of AβPP-Aβ42 should be neuroprotective as AβPP-Aβ42 is the neurotoxic form of the secreted peptide. In a controlled clinical trial, ApoE-ϵ4-negative individuals were demonstrated to benefit significantly from intranasal insulin, as manifested by improvements in cognitive performance [308]. The fact that ApoEϵ4+ subjects did not benefit from the same treatment suggests that intranasal insulin, as well as other pro-metabolic therapies for AD, may have to be tailored according to particular genetic risk factors and biomarkers of disease. 

## INSULIN STIMULATING/RELEASING HORMONES (INCRETINS) 

As an alternative to insulin, another promising approach is therapeutic administration of incretins, such as glucagon-like peptide-1 (GLP-1). GLP-1 is an insulinotropic peptide that is generated by cleavage of proglucagon protein, and secreted by small intestinal L cells following food intake. GLP-1 has a half-life of only a few minutes, and is rapidly degraded by dipeptidyl peptidase-4. GLP-1 stimulates insulin gene expression and secretion, and suppresses glucagon. GLP-1 lowers blood glucose in individuals with T2DM [309, 310], and it restores insulin sensitivity. The latter attribute is perhaps one of the most important considerations for the potential use of GLP-1 and related molecules for the treatment of brain insulin resistance in AD. 

Like insulin, GLP-1 stimulates neuritic growth in CNS neurons and exerts neuroprotecive actions against glutamate-mediated excitotoxity, oxidative stress, trophic factor withdrawal, and cell death [311-313]. In addition, inhibition of dipeptidyl peptidase-4, which degrades GLP-1, reduced oxidative and nitrosative stress, inflammation, memory impairment, and AβPP-Aβ deposits in an AD transgenic mouse model [314]. At the very least, these observations support the hypothesis that insulin resistance and deficiency play critical roles in the pathogenesis of AD. Importantly, GLP-1 can cross the blood-brain barrier, and may effectively reduce brain AβPP-Aβ burden in AD [309, 310, 315]. With the realization that GLP-1 has a short half-life and therefore limited practical use for long-term therapy, synthetic long-lasting analogues of GLP-1 have been generated and proven to be effective in preserving cholinergic neuron function [316]. The development of GLP-1 receptor agonists, such as Geniposide or Exendin-4, which harbor the same neuro-protective and neuro-stimulatory properties as GLP-1 [317], but have longer half-lives [311, 315, 318, 319], may provide effective and standardized long-term options for treating brain insulin resistance diseases such as AD. Finally, a future approach could be to utilize a form of gene therapy in which genetically modified mesenchymal or stem cells are implanted into the lateral ventricles for sustained delivery of neuro-stimulatory and neuro-protective agonists [320-322], including GLP-1 [323].

### Anti-Hyperglycemic Agents

Metformin is a biguanide anti-hyperglycemic drug that is used to treat T2DM. Metformin functions by suppressing gluconeogenesis and enhancing glucose uptake and insulin sensitivity. Metformin treatment is protective against neurological complications of T2DM, including cognitive impairment and cerebral vascular disease [324]. Although metformin treatment was found to increase the generation of both intra- and extracellular AβPP-Aβ due to increased expression of BACE1, suggesting that metformin and insulin may have opposing effects on AβPP-Aβ accumulation, the administration of both insulin and metformin provided significant neuroprotection. Importantly, the combined treatments reduced AβPP-Aβ levels, the severity of AD pathology, including AβPP-Aβ neuritic plaques, and oligomeric AβPP-Aβ-mediated down-regulation of the insulin receptor. These findings suggest that metformin mono-therapy may be harmful due to exacerbation of AD-type neurodegeneration [325], whereas the combined use of insulin and hypoglycemic drugs may benefit elderly patients in the early stages of AD by significantly improving cognitive performance and slowing the rate of neurodegeneration. 

### Insulin Sensitizers

Peroxisome proliferator-activated receptors [PPAR] are steroid hormone super family ligand-inducible transcription factors that enhance insulin sensitivity, modulate glucose and lipid metabolism, stimulate mitochondrial function, and reduce inflammatory responses [326-329]. Three classes of PPARs are recognized, PPAR-α, PPAR-δ, and PPAR-γ. All 3 are expressed in the adult brain, although PPAR-δ is most abundant, followed by PPAR-γ [8, 83, 149]. PPAR agonist treatments improve cognitive performance in experimental animal models [83, 330] and in humans with AD or MCI [148, 150, 331]. The PPAR-γ agonist, rosiglitazone, has been most widely studied in human clinical trials. In addition to its insulin sensitizing and anti-inflammatory properties, rosiglitazone, like metformin, increases expression of the GLUT4 glucose transporter and glucose metabolism. Moreover, simultaneous treatment with PPAR agonists such as, rosiglitazone, enhances the therapeutic effects of metformin+insulin. 

In a small double-blind, placebo-controlled trial, investigators showed that rosiglitazone treatment significantly preserved performance on delayed recall and attention tasks relative to the placebo-treated group, which continued to decline [332]. However, a later study found that rosiglitazone therapy was mainly effective in preserving cognition in patients who were ApoE ϵ4-negative, while the ApoE ϵ4+ subjects showed no improvement or continued to decline [333]. Despite promising experimental results, initially positive clinical studies, and supportive evidence that impaired glucose metabolism and insulin resistance are key components in the pathogenesis of AD, the most recent outcome of a rosiglitazone monotherapy, randomized double-blind placebo controlled phase III study was negative with respect to improvements in objective cognitive assessments, but highly statistically significant based on clinical and caregiver impression [334]. Potential explanations for these disappointing results include the following: 1) effective treatment of neurodegenerative diseases may require a different isoform of PPAR agonist, i.e. PPAR-δ, since PPAR-δ is abundantly expressed in the brain, and previous studies showed that PPAR-δ agonist treatment more effectively prevented AD-type neurodegeneration and neurocognitive deficits compared with PPAR-α and PPAR-γ agonists [83]; 2) the biodistribution of the PPAR agonists may not have been optimized based on the structure of the compounds; and 3) mono-therapy may not be sufficient, and instead the combined administration of a PPAR agonist with insulin or GLP-1 and metformin may be required to effectively treat AD-associated brain insulin resistance and metabolic dysfunction.

#### Alpha Lipoic Acid (ALA)

ALA is a natural compound that supports mitochondrial function, serving as a cofactor for pyruvate dehydrogenase and alpha ketoglutarate dehydrogenase. Importantly, ALA enhances production of acetylcholine by activating choline acetyltransferase and increasing glucose uptake [335]. Therefore, the potential benefits of ALA are mediated by the supportive actions of ALA on insulin and GLP-1. However, beyond those effects, ALA has anti-oxidant effects, since it serves as an inhibitor of hydroxyl radical formation and can scavenge reactive oxygen species and lipid peroxidation products such as 4-hydroxy-2-nonenal [335], which are increased in AD brains [336, 337]. In addition, ALA inhibits expression of pro-inflammatory cytokines and inflammation-associated nitric oxide synthase, which have important roles in mediating neuro-inflammation in the early stages of AD [338-341]. Although there are few clinical trials examining the efficacy of ALA therapy for AD, there is some evidence that ALA may slow the progression of cognitive impairment in patients with moderately severe AD [335]. 

#### Chromium Picolinate

Chromium is an often overlooked essential metal that has an important role in regulating the actions of insulin, including carbohydrate, protein, and lipid metabolism [342-344]. Chromium enhances insulin sensitivity by increasing insulin receptor binding, insulin receptor number, and insulin internalization. In addition, chromium lowers blood glucose, triglycerides, and low density lipoprotein (LDL) cholesterol, increases high density lipoproteins (HDL), and reduces risk for cardiovascular disease [345, 346]. Moreover, chromium supplementation improves cognitive performance, including memory, promotes weight loss, and helps control diabetes. Chromium picolinate mediates its effects on body weight by reducing food craving and increasing satiety [347]. Although food sources of chromium are fairly abundant and include, whole grains, lean meats, cheeses, corn oil, black pepper, thyme, and brewer’s yeast, most foods contain relatively low levels of chromium per serving (1-2 mcg), and most dietary forms of chromium are poorly absorbed. On the other hand, chromium picolineate, which is highly stable and consists of Cr[III] chelated with three molecules of picolinic acid, was formulated to increase the bioavailability of ingested chromium [346]. *In vivo* studies have demonstrated considerable safety associated with chromium picolinate use, including long-term exposures [348, 349]. 

Although clinical studies investigating the therapeutic effects of chromium picolinate supplementation have yielded conflicting results, in the vast majority of clinical trials (utilizing 150-1000 mcg/day), chromium picolinate was found to significantly improve glycemic control and reduce blood cholesterol and triglyceride levels in diabetics [350]. With increasing age, plasma chromium levels decline. This phenomenon could partly account for aging-associated insulin resistance and cognitive decline since reduced levels of chromium are correlated with cognitive impairment and AD [351]. In a recent double-blind placebo controlled clinical study, a 12-week period of chromium picolinate supplements administered to elderly subjects significantly reduced semantic interference on learning, recall, and recognition memory tasks [352]. In addition, functional magnetic resonance imaging demonstrated increased activity in the thalamus, and frontal, parietal and temporal lobes of treated subjects, indicating that chromium picolinate can enhance cognitive function in elderly people [352]. Due to its positive effect on insulin signaling mechanisms and relative safety, it appears that chromium supplementation has a role in long-term support of metabolic pathways, both systemically and within the CNS. Given the demonstrated roles of T2DM, obesity, and peripheral insulin resistance as mediators of cognitive impairment and neurodegeneration, and supportive evidence that improvements in insulin responsiveness enhance cognitive function and reduce neurodegeneration, the inclusion of chromium picolinate as a dietary supplement, or development of like compounds that achieve the same, or better and more reproducible effects could help target fundamental metabolic dysfunctions that occur early in the course of AD. 

### Antioxidant and Anti-Inflammatory Drugs

Antioxidant functions help maintain mitochondrial homeostasis, neuronal activities, and cell survival. Oxidative stress plays a pivotal role in the pathogenesis and progression of AD-type dementia. Sources of oxidative stress include, impairments in insulin signaling, fibrillarization of oligomeric AβPP-Aβ and tau, mitochondrial dysfunction, micro-vascular disease, increased accumulation of ROS, RNS and inflammation [353]. Although it has not yet been determined which source of oxidative stress in most critical to neurodegeneration and cognitive impairment, some doubt has been cast upon the role of AβPP-Aβ since in a longitudinal analysis, significant reductions in plasma AβPP-Aβ42 in subjects treated with various anti-inflammatory agents, was not associated with improvements in cognition [354]. Nonetheless, the interest in reducing oxidative stress in the brain is justified as a treatment approach because this type of injury could, at the very least, serve as a co-factor mediating AD progression. Potential approaches to reduce oxidative stress include the use of anti-oxidants, anti-inflammatory agents, radical scavengers, transition metal chelators, and non-vitamin anti-oxidant polyphenols.

#### Vitamin E [Tocopherol] 

It has anti-oxidant properties, and has been shown to modulate signaling and aid in neurotransmitter synthesis. However, epidemiological data linking regular use of vitamin antioxidants to cognitive performance are weak, and although prospective longitudinal and interventional studies have shown some positive results, the responses detected in randomized control studies have been modest [355, 356]. On the other hand, discrepancies between clinical trials and epidemiological data could be explained by the relatively short periods of follow-up needed for a longitudinal study to be practical, versus retrospective analysis of possibly life-long exposure effects. The main conclusion that can be drawn from the available data is that antioxidant therapy, while beneficial, cannot be used as a stand-alone treatment for AD, but its use should probably be incorporated into lifestyles. 

#### Non-Steroidal Anti-Inflammatory Drugs (NSAIDs)

Based on epidemiologic studies demonstrating apparently reduced risk of developing AD among primarily ApoEϵ4+ individuals who had been chronically treated with NSAIDs, it was hypothesized that NSAIDs would have efficacy in treating AD, or preventing AD development in patients with MCI [79, 284, 357-359]. These concepts are supported by the fact that neuro-inflammatory responses occur early in the course of AD, and they contribute to AβPP-Aβ deposition [360]. In addition, in experimental models, neuro-inflammation leading to the recruitment and activation of microglia and astrocytes, mediates AβPP-Aβ deposition [361]. It was hypothesized that NSAIDs could be used to modify AD pathogenesis by suppressing neuro-inflammation and slowing AβPP-Aβ deposition [359, 362, 363]. However, in clinical trials, selective cyclooxygenase-2 (COX-2) inhibitor drug therapy proved to be ineffective for treating AD [284, 358], and protecting individuals with MCI from progressing toward AD [284]. These observations make it unlikely that this avenue of therapy will be useful for modifying the course of AD.

#### Radical Scavengers

Epidemiological studies suggested that long-term treatment with anti-inflammatory drugs, including NSAIDs, vitamin E, estrogens, and 3-hydroxy-3-methyl-glutaryl-CoA (HMG-CoA) reductase inhibitors (statins) might be neuroprotective in either preventing dementia, or improving clinical outcomes [265]. The potential benefits and limitations of NSAIDs and Vitamin E therapy for AD have already been discussed. Interest in the role of estrogens was inspired by the findings that, estrogens stimulate cognitive performance in experimental animals and, bio-available estrogen levels decline with advancing age in both sexes [364, 365], Although a few clinical studies showed limited and mainly short-term rather than long-term benefits of estrogen therapy with respect to cognition [365], better controlled clinical trials provided clear evidence that exogenous estrogen therapy does not improves dementia symptoms in women with AD, and instead, it increases dementia risk when the treatment is begun after 65 years of age [366, 367]. On the other hand, the recent evidence that estrogen receptor modulation therapy may improve cognition [364, 368] deserves further study. 

Statins are HMG-CoA reductase inhibitors. HMG-CoA catalyzes the rate-limiting step in cholesterol biosynthesis. The rationale for using statins to treat AD is that cholesterol metabolism and transport are involved in the regulation AβPP-Aβ deposition and tau hyper-phosphorylation [369, 370]. In addition, cerebrovascular disease, which can cause vascular dementia and contribute to AD progression, is associated with hypercholesterolemia. Statin therapy has been evaluated in clinical trials, and meta analysis of data generated by large prospective clinical trials revealed no significant benefits of atorvastatin or simvastatin therapy in patients with dementia who had been treated for periods ranging from 26 to 72 weeks, despite significant reductions in serum low density lipoprotein (LDL) [371-374]. Still, other studies showed significant reductions in incident dementia among statin users [375, 376] and experimental data suggests that statins may provide some degree of neuroprotection [377]. 

In an anti-inflammatory treatment prevention trial, despite a 67% reduction in hazard risk of incident AD among individuals treated with lipid-lowering drugs, the most significant findings were that HDL was positively correlated with mini-mental state examination (MMSE) performance, and while LDL cholesterol was negatively correlated with immediate and delayed recall [378]. Limitations of this study include, its relatively short duration of follow-up and the lack of distinction between vascular dementia and AD. However, the impact of statin therapy was most likely effectuated by reduced severity of cerebrovascular disease, lessening its contribution to AD progression. Potential concerns over the use of statins to treat AD have been raised by the findings that: 1] brain cholesterol levels are reduced in AD [370]; 2] reductions in neuronal cholesterol lead to impaired insulin signaling and energy metabolism [49]; and 3] cognitive impairment can occur with chronic statin use [379-382] and following its discontinuation, cognitive function may be restored [380, 382]. These observations suggest that routine and preventive use of statin therapy, particularly in the elderly, should be re-evaluated [383] and perhaps avoided unless indicated for cardiovascular health. Moreover, future studies should assess risk for further cognitive impairment among individuals with AD who do not have hyperlipidemia or cerebrovascular disease. 

#### Ginkgo Biloba

Ginkgos or Maldenhair Trees, are ancient and have served as sources of food and traditional medicine for centuries. Gingko biloba trees are commonly cultivated in China, Japan, and North America. Gingko nuts are used in congee, and the leaf extracts contain medicinal flavonoids and terpenoids. Gingko is thought to have neurotropic properties and enhance memory and concentration, suggesting it may be useful for treating or preventing AD. Clinical trials designed to examine the therapeutic efficacy of gingko biloba have mainly used the leaf extract, EGb 761, which contains 24% flavone glycosides and 6% terpenoids. Experimentally, EGb 761 enhances antioxidant capacity of cells by inducing expression of the glutathione synthesis catalytic subunit, GCLC [384]. In humans, EGb761 significantly increases regional cerebral blood flow [385], suggesting that its therapeutic effects may be mediated by enhanced perfusion.

Although short-term effects of Gingko biloba therapy on cognitive function have been reported [386], several studies, including a community-based pragmatic randomized double-blind study, detected no significant halting or slowing of cognitive decline occurred after a 6-month trial with standard Gingko biloba extract [387]. On the other hand, in a 42-month randomized placebo-controlled, double-blind study, after correcting for medication adherence, significant protective effects of gingko biloba extract (EGb 761), manifested by slower rates of cognitive and memory decline, were observed [388]. In another randomized control trial in which subjects were administered 240 mg/day of EGb 761 for 22 weeks, significant improvement in cognitive performance was also observed in patients with AD or vascular dementia relative to placebo-treated patients [389]. Two independent meta analyses analyzing the effectiveness of EGb761 administration in 6 months or longer duration clinical trials demonstrated statistical trends corresponding to positive or protective effects of EGb761 versus placebo, although effect sizes were estimated to be moderate [390, 391]. 

Despite discrepancies in study outcomes and conclusions due to underpowered designs and short-term follow-up intervals, altogether the data suggest that Ginkgo biloba has mild to moderate neuroprotective effects in both AD and vascular dementia, and that it may also benefit elderly individuals with MCI. The recent meta analyses reports described above are encouraging, and support the concept that antioxidants in general, and Ginkgo biloba specifically, may be beneficial in the therapeutic management of cognitive impairment and AD. Importantly, there are no demonstrated safety concerns associated with the standard EGb 761 extract dose of 240 mg/day [392]. The therapeutic mechanisms of action are related to improvements in cerebral blood flow and antioxidant properties of the compound. 

#### Transition Metal Ion Chelators

One hypothesis that has remained viable for decades is that transition metal ions, including Al (III), Fe (III), Zn (II), and Cu (II), cause neurotoxicity and mediate neurodegeneration [393-395], even in the earliest stages of AD [396]. Excess accumulation of transition metal ions promotes oxidative stress, apoptosis, and aggregation and fibrillarization of hyper-phosphorylated tau [397] and AβPP-Aβ42 [395, 398]. Oxidative stress is mediated by the formation of hydroxyl radicals following interactions between iron and hydrogen peroxide. In AD, brain levels of free heme and hemin are significantly elevated [399], and probably contribute to neurodegeneration by inhibiting cholinergic function, altering AβPP-Aβ metabolism, binding to hyper-phosphorylated tau and promoting tau aggregation into paired-helical filaments, and inducing formation of free radicals [399]. 

Chelation with compounds such as desferrioxamine, Feralex-G, or Clioquinol affords neuroprotection by preventing the aggregation and fibrillarization of AβPP-Aβ and tau, and reducing ROS production [397, 400-402]. Correspondingly, Clioquinol chelation was reported to reduce AβPP-Aβ burden in transgenic mice [400, 401]. Mechanistically, in addition to its proposed direct anti-aggregation effects on AβPP-Aβ, chelation therapy could reduce AβPP-Aβ deposition by decreasing oxidative stress and ROS [77], which could be caused by heme and heavy metals. Chelation therapy for AD has also been tested in humans. In a 2-year long randomized placebo-controlled trial of twice daily injections of the trivalent chelator, desferrioxamine, the rates of performance decline among patients with probable AD slowed significantly [403]. However, in a later uncontrolled clinical trial of Clioquinol therapy for AD, the subjects showed only modest improvements in clinical ratings [402]. In one case of familial AD, clioquinol therapy increased cerebral glucose metabolism and stabilized clinical status [404]. Only a few studies have linked chelation therapy to improved glucose utilization, energy metabolism, and insulin signaling in the brain. Nonetheless, the findings that chelation of zinc and iron prevents or attenuates streptozocin-, alloxan-, or ferritin-induced diabetes [405-407], and that desferrioxamine chelation of iron, and dietary restriction of iron increase glucose uptake and insulin signaling in hepatocytes [408, 409] are intriguing with respect to the roles of brain insulin resistance and metabolic dysfunction in the pathogenesis of AD and neurodegeneration. Since treatment with antioxidants, Vitamin E, Vitamin C, Heme oxygenase 1, or metal chelators prevents the neurotoxic effects of heme and hemin [410], and may also enhance insulin signaling and glucose utilization in the brain, heme-induced oxidative stress could potentially be targeted by anti-oxidant and chelation therapy to help restore cholinergic function, reduce fibrillarization of tau and AβPP-Aβ42, decrease oxidative stress, and improve energy metabolism in the brain.

Despite probable benefits, a major limitation of our current methods of chelation therapy is that delivery of drugs with high Fe (III) binding capacity to the CNS are suboptimal [411, 412]. Another point is that caution must be exercised regarding the potential liberal use of chelation therapy because iron is needed to generate energy, and copper, manganese, and zinc participate in enzymatic pathways that protect cells from free radicals and reactive oxygen species, as they are functionally required for the activation of superoxide dismutases I-III. Therefore, thorough removal of these metal ions would be detrimental. To address these problems, various compounds have been developed and tested in pre-clinical models. For example, DP-109 is a lipophilic metal chelator that was demonstrated to reduce cerebral AβPP-Aβ burden in Tg2576 transgenic mice [413]. Another approach considered was to conjugate chelators to nanoparticles that can cross the blood-brain barrier to chelate metal ions, and then exit to remove them [414-416]. Recently, Nano-N2PY, a prototype nanoparticle-chelator conjugate was demonstrated to inhibit AβPP-Aβ aggregation and reduce AβPP-Aβ-associated cortical neuron toxicity *in vivo* [417]. Another novel approach involved the development of site-activated multifunctional chelators, such as HLA20A, that become activated by binding and inhibiting acetylcholinesterase, resulting in the release of an active chelator that reduces AβPP-Aβ fibrillization and oxidative stress [418, 419]. Along related lines, dual target-directed 1,3-diphenylurea derivatives seem capable of both inhibiting BACE1 and chelating metal ions [420].

#### Polyphenols [Red Wine, Green Tea, and Curcumin]

Epidemiological studies demonstrated relative protection from dementia, AD, and Parkinson's disease among individuals who regularly consumed green tea or red wine [421]. Resveratrol, 3.4',5-trihydroxy-trans-stilbene, is a natural polyphenol that is abundantly present in red wine and has antioxidant and neuroprotective activities. Besides red wine, other substances such as grape seed extracts, contain resveritrol and therefore also provide neuroprotection [422, 423]. Pharmacokinetic studies have affirmed that grape seed polyphenols abundantly distribute to the brain [424]. Experimentally, resveratrol's neuroprotective effects are mediated by enhancement of glutathione free radical scavenger activity [425, 426], and reduction in AβPP-Aβ levels [427] due to increased clearance via the proteasome [428] or autophagy and lysosomal degradation [429]. Resveratrol also exerts cytoprotective effects by stimulating heme oxygenase, and modulating cellular resistance to insults such as compromised blood flow, injury, and inflammation [430]. In addition, resveratrol and other polyphenols are effective metal chelators, and protect the brain from oxidative stress and ROS generation caused by accumulations of lead, iron, aluminum, zinc, and copper [431].

One very interesting effect of resveratrol is its ability to retard aging and protect against AD due to stimulation of the sirtuin protein, SIRT1 [432]. Sirtuin genes promote longevity, and SIRT1-mediated deacetylase activity protects against AD-type neurodegeneration [433, 434]. Mechanistically, SIRT1 functions by interfering with AβPP-Aβ peptide generation [433, 434], and SIRT1-activating molecules such as resveratrol, were shown to reduce neurodegeneration and prevent learning impairments in the p25 transgemic mouse model of AD, which is associated with tau hyper-phosphorylation and fibrillarization [435]. Importantly, SIRT1 activation achieves the same effect as caloric restriction with respect to preventing aging and AD [436]. Caloric restriction with weigh loss is a well-established means of increasing insulin sensitivity, and it works by activating the forkhead transcription factor, FoxO3a, which is a key regulator of insulin and IGF-1 signaling [437]. In AD brains, FoxO3a activity is reduced [437], corresponding with the impairments in insulin and IGF-1 signaling. 

The major green tea polyphenolic compound, epigallocatechin-3-gallate (EGCG), has neuroprotective actions similar to those of resveritrol. Studies have shown that EGCG: 1) mimics cellular effects of insulin, reducing gluconeogenesis and corresponding enzyme gene expression [438]; 2) reduces AβPP-Aβ levels by enhancing cleavage and clearance of the C-terminal fragment of AβPP [439]; 3) functions as an iron chelating and mitochondrial stabilization compound [440, 441]. Moreover, clinical trials have demonstrated that EGCG has neuroprotective and anti-oxidant therapeutic effects in AD, as well as Parkinson's disease [439, 441]. To circumvent problems related to dosing and CNS delivery, nanolipidica EGCG particles have been generated and already shown to improve brain distribution following oral administration [442]. 

Curcumin [tetrahydrocurmin], a yellow polyphenol and the active component in tumeric, functions as an anti-diabetic, anti-oxidant, anti-lipidemic, anti-inflammatory compound [443-448], similar to the effects of resveratrol and EGCG. Curumin induces glucose uptake through phosphorylation of AMP kinase (AMP-activated protein kinase /acetyl-CoA carboxylase) and GLUT4 translocation to the cell surface. Curcumin also enhances insulin sensitivity, resulting in synergistic activation of the AMPK and PI3K/Akt pathways [449]. In addition, curcumin increases pancreatic islet cell insulin secretion, possibly through increased expression of heme-oxygenase 1 [450]. Even in established cases of diabetes mellitus, curcumin has demonstrated hypoglycemic effects via reversal of gluconeogenic enzymes, reducing levels of glycosylated hemoglobin (HbA(1C)), and increasing plasma insulin, glycogen, and C-peptide [451, 452]. Recent studies showed that curcumin significantly increases expression of GLUT3, acetylcholine receptors, and insulin receptors [453], and decreases lipid peroxidation [454] in brains of experimental animals with streptozocin-induced diabetes, and neurodegeneration. Together, the data point favorably toward the neuroprotective, anti-aging, and anti-oxidant effects of polyphenols. In addition, the findings demonstrate that polyphenols mediate their effects by enhancing insulin and IGF signaling, and quite likely stimulate sirtuin signaling, which accomplishes all of the aforementioned therapeutic effects of polyphenols. In addition, the aggregate results give strong support to the concept that impaired brain insulin/IGF signaling and energy metabolism play pivotal roles in the pathogenesis of AD. This leads to the obvious conclusion that, in addition to multi-modal pharmaceutical targeting of brain insulin/IGF resistance, oxidative stress, inflammation [metal ion accumulation], and AβPP-Aβ and tau misfolding and fibrillarization, lifestyle measures could be effective in helping to prevent or delay the onset of aging-associated neurodegenerative diseases, including AD. 

### Diet, Supplements, and Lifestyle

There is little doubt that the explosion in insulin resistance diseases, including diabetes, obesity, non-alcoholic fatty liver disease, polycystic ovarian disease, and dementia, followed revolutionary changes in the Western diet that resulted in an exponential rise in the consumption of highly processed foods that either lack the appropriate nutrients for long-term support of CNS and systemic metabolic functions, or contain added ingredients that impair protective actions of natural food substances [455]. Until recently, long-term pro-active nutritional support has taken a back seat with respect to disease prevention, particularly among allopathic physicians. In retrospect, the concept is illogical given the clear evidence that: 1) certain amino acids help elaborate neurotransmitters and trophic factors for brain function; 2) omega-3, other essential fatty acids, and Vitamin E (alpha tocopherol) [456] support membrane maintenance and renewal needed for neuronal plasticity; 3) Vitamins A, C, E, and beta carotene have anti-oxidant properties; and 4) many B vitamins, including Vitamins B1 (thiamine), B2 (riboflavin), B3 (niacin), B6 (pyridoxine), B9 (folate), and B12 (cobalamin) are required for a broad range of neurological functions, including cognition [457]. 

Omega-3-fatty acids function as ROS scavengers, and they are essential for brain growth and function throughout life. For example omega-3 fatty acids modulate various neuronal functions, protect against neuronal oxidative stress, and inhibit signaling pathways that promote tau phosphorylation and assembly into paired helical filaments [458]. Aging-associated cognitive impairments have been linked to omega-3-fatty acid deficiencies, while dietary supplementation with docosahexaenoic acid (DHA), which is the major form of omega-3 fatty acid found in neurons and available in the diet from oily fish [459], was shown to be neuroprotective in lowering the risks of cognitive impairment and AD [458, 459]. Observational studies provide positive evidence that chronic omega-3 ingestion is beneficial to cognitive function [458, 460, 461], while low fish consumption increases risk for AD [462]. Experimentally, eicosapentaenoic acid (EPA) and DHA reduce AβPP-Aβ fragment formation, and DHA enhances synapse formation [463]. Although observational and epidemiological studies positively support the concept that omega-3 and other essential fatty acids should be regularly incorporated into the diet, specific guidelines regarding dosages and frequency are difficult to establish because firm conclusions can seldom be drawn based on short-term clinical trials [460]

With regard to the B vitamins, thiamine (B1) is needed for glucose metabolism and energy production, and thiamine deficiency syndromes include psychosis, dementia, peripheral neuropathy, and heart failure. Folate (B9) preserves memory during aging, and when combined with vitamin B12, delays the onset of dementia. Deficiencies in pyradoxine [B6] and cobalamin [B12] are correlated with cognitive impairment. In addition, B12 negatively regulates plasma homocysteine, and high levels of homocysteine increase risk for cardiovascular disease, cerebrovascular disease, and cognitive impairment. In short, deficiencies in B vitamins lead to chronic disablement of insulin signal transduction pathways, neurotransmitter functions, and cognition. Although many studies designed to objectively examine the therapeutic effectiveness of vitamin and nutritional supplements for preventing neurodegeneration have failed, conclusions drawn from their negative results should be cautious because for the most part, the study designs tend to be either underpowered or too limited in duration to achieve statistically significant results [464]. Conceivably, population based epidemiologic studies, combined with experimentation, may provide the best guidance for the long-term use of dietary supplements for brain health.

Based on data culled from a broad range of studies on the effectiveness of lifestyle and dietary measures for supporting brain health and providing some degree of neuroprotection, the following conclusions could be drawn: 1) aerobic exercise and caloric restriction significantly improve cognitive performance and slow progression toward neurodegeneration. These effects are mediated by enhancement of insulin and IGF responsiveness and slowing of the aging process, in part due to SIRT1 activation. Aging is by far the most significant risk factor for AD [434, 436, 465-470] and AD is mediated in large measure by brain insulin resistance and impaired energy metabolism [7, 8]. Therefore, measures that support or bolster insulin sensitivity, including exercise, caloric restriction, and loss of excess weight retard both aging and lower the risk of AD; 2) dietary supplements such as omega-3 fatty acids and compounds such as chromium picolinate, curcumin, alpha lipoic acid, cinnamon, and red bean lectin (stimulates GLP-1), that have insulin sensitizing properties [471], could help support neuronal plasticity and signaling mechanisms that regulate neuronal metabolism and reduce fibrillarization of AβPP-Aβ and tau [394]; 3) agents that reduce oxidative stress, including fat soluble vitamins, natural statins such as those supplied in red yeast rice, and polyphenols, including those present in red wine, green tea, curcumin, and soy isolate protein [472], reduce the generation of reactive oxygen and reactive nitrogen species, DNA damage, and activation of pro-inflammatory, pro-injury, and pro-death signaling; 4) ample supplementation with multiple B vitamins that support nervous system and cardiovascular function, and reduce homocysteine levels [473] are neuroprotective. 

In summary, a vast volume of literature has been summarized and discussed in an attempt to demonstrate how seemingly disparate concepts and opinions about the mechanisms of neurodegeneration actually converge toward a relatively recently appreciated theme that impairments in brain insulin and IGF signaling and responsiveness are at the core of AD. Insulin and IGF resistance can account for the deficits in brain glucose utilization and energy metabolism that are detectable early in the course of AD. The attendant inhibition of insulin/IGF signaling leads to aberrant activation of kinases that lead to tau hyper-phosphorylation. Impairments in energy metabolism and glucose utilization have broad consequences due to increased oxidative stress, activation of pro-inflammatory cascades, and ROS generation, all of which promote aberrant AβPP expression and cleavage, AβPP-Aβ42 accumulation, and fibrillarization and misfolding of tau and AβPP-Aβ. Increased ROS production causes electrophilic attacks on proteins, lipids, and nucleic acids, resulting in the formation of adducts that promote further structural and functional damage, oxidative stress, ubiquitination of proteins, targeting them for degradation. Insulin/IGF resistance impairs lipid metabolism, leading to disruption of myelin homeostasis. AD is associated with white matter atrophy, myelin loss, and increased myelin breakdown with production of potentially toxic sphingolipids, including ceramides. Neurotoxic ceramides promote insulin resistance, neuroinflammation, and oxidative stress. Finally, brain insulin/IGF resistance can also explain the frequent co-existence of cerebral microvascular disease, which substantially contributes to the neuropathology of AD. 

Genetic or familial forms of AD represent the minority of the overall population at risk for developing AD. Although valuable lessons have been and will continue to be learned by studying genetic forms of this disease, future efforts should be focused on understanding factors that contribute to the pathogenesis and progression of sporadic AD. Certainly the extremely rapid rise in AD prevalence rates, manifested by up to several hundred-fold higher age-adjusted rates in 2005 compared with 1980 cannot be explained by genetic factors, and instead parallels trends that characterize exposure models of disease [1]. In fact, the age-adjusted trends in AD prevalence rates are similar to those observed for diabetes mellitus [1]. Although we do not know the cause[s] of AD, epidemiological, observational, and experimental evidence together support the hypothesis that AD is a metabolic disease with virtually all of the features of diabetes mellitus, but largely confined to the brain. One very important conclusion that could be drawn from this review is that the concept of using mono-therapy to treat AD is wrong, and instead, multiple targets must be attacked simultaneously and over a prolonged period of time [260, 474], similar to current approaches used to treat malignancies. Future multi-modal therapies for AD should be directed at multiple levels of demonstrated weakness within the insulin/IGF signaling cascade, beginning with receptor sensitizers, agents to promote insulin production and release, e.g. GLP-1, inhibitors of oxidative stress, radical formation, and metal ion accumulation, tau phosphorylating kinase modulators, and co-factors that support glucose utilization, mitochondrial function, and energy metabolism. If effective, these combined treatments will likely enhance neurotransmitter activity and availability, neuronal plasticity, and neuronal survival, which are needed to preserve cognitive function.

Complementary and alternative medicine approaches have been used extensively and for centuries throughout the world. The institution of modern medicine has concerns and reservations about embracing the philosophies of naturopathic, homeopathic, and complementary and alternative medicine because allopathic medicine is evidence-based, i.e. based on objective scientific and clinical experimentation. As modern medicine advances scientifically, personalized diagnostics and therapeutics will continue to grow more mechanized, molecular, biochemical, and genetic test-driven, and algorithm-based. However, the unintended consequences include, rank dismissal or abandonment of common-sense preventive and counseling approaches. Unfortunately, we are still without effective means to accurately detect and characterize AD in its early stages, when it would be most responsive to treatment, and we lack effective and universally accepted long-term approaches to treatment and prevention of AD, despite enormous effort and funds spent over the past several decades. Inconsistencies among studies designed to evaluate the effectiveness of natural compounds for treating or preventing neurodegeneration stem from observational versus double-blind placebo controlled clinical trials, with generally more favorable data obtained from the former [475].

Frustration over the lack of answers and sustained positive outcomes from state-of-the-art therapy, has probably helped to fuel the growing utilization of complementary and alternative medicine to treat AD. Instead of focusing on the use of pharmaceutical grade, FDA-approved drugs, emphasis is placed on lifestyle modifications, diet, macronutrient and micronutrient supplements, and consumption of natural compounds to prevent, retard, or cure chronic diseases [476]. These approaches are generally dismissed outright, or met with heavy skepticism due to the lack of clear and systematic guidelines for evaluating effectiveness of complementary and alternative therapies for improving cognitive performance [477]. Moreover, although specific nutritional deficiencies have been correlated with particular disease states, growth in our knowledge of how micro- and macronutrients contribute to brain and bodily health, and how they may prevent, delay, or modify the course of chronic disease, has been slow. Finally, many studies designed to objectively examine the effectiveness of these alternative approaches have been either underpowered or too limited in duration to achieve statistically significant results, leading some to conclude that such measures would be fruitless [464]. Conceivably, population based epidemiologic studies, combined with experimentation, may provide the best guidance in the long-term use of dietary supplements. 

## Figures and Tables

**Fig. (3):**
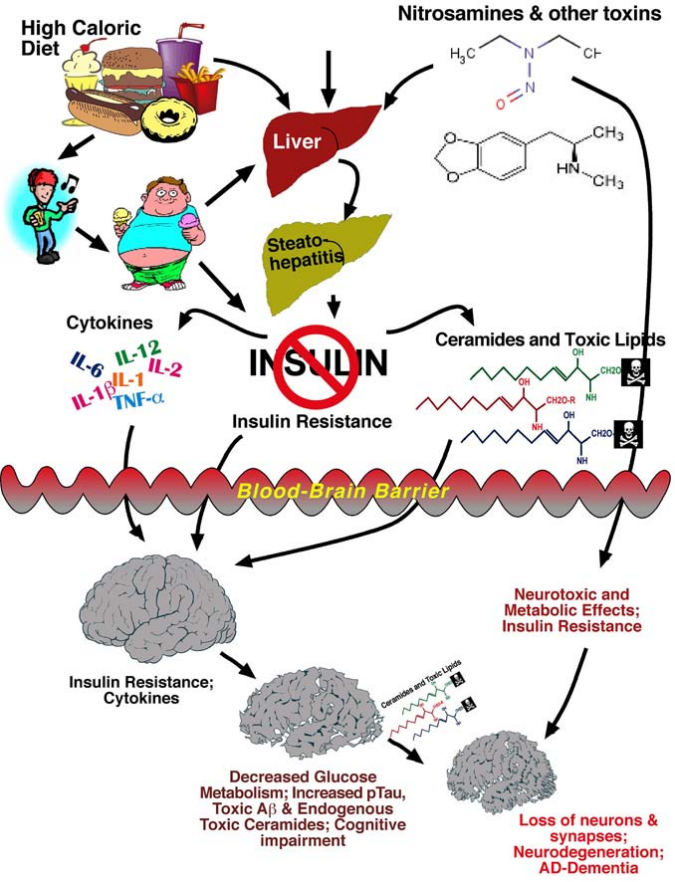
High caloric intake and/or chronic low-level nitrosamine exposures [through diet, smoking, agriculture], promote fatty liver disease (steatohepatitis) that progresses due to injury and inflammation, eventually leading to hepatic insulin resistance. The same poor physiological states also promote obesity, diabetes mellitus, and other peripheral insulin resistance diseases. Toxic lipids, including ceramides, made in the liver, get released into the circulation, cross the blood-brain barrier, and cause brain insulin resistance, inflammation, energy failure, toxicity, and local production of toxic ceramides. The end result is progressive neurodegeneration, including Alzheimer’s disease.

**Table 1.** Metabolic Hypothesis of Alzheimer's Disease-Consequences of Brain Insulin Resistance

Impairment	Adverse Effect	Role in Alzheimer's Disease
GLUT4 function	Reduced glucose uptake and utilization	Energy deficits; compromised homeostatic functions, disruption of neuronal cytoskeleton, synaptic disconnection
Insulin receptor function	Decreased signaling through IRS, PI3K-Akt	Reduced neuronal and oligodendroglial survival, neuronal plasticity, myelin maintenance
	Increased activation of GSK-3β and phosphatases that negatively regulate insulin signaling	Increased tau phosphorylation, oxidative stress, neuro-inflammation, pro-apoptosis signaling Decreased Wnt signaling
	Reduced insulin-responsive gene expression	Reduced choline acetyltransferase expression --> deficits in acetylcholine Decreased GAPDH expression, further impairment of glucose metabolism
Insulin receptor function or hyper-insulinemia	Endothelial cell injury, intimal thickening, and vessel wall fibrosis	Microvascular disease and cerebral hypoperfusion
Mitochondrial function	Increased oxidative stress, ROS, RNS	DNA damage, lipid peroxidation, energy deficits, cell death, increased AβPP expression, Aβ42 deposition and fibrillarization
Myelin maintenance	Myelin breakdown, increased generation of ceramides and other toxic sphingolipids; lipid peroxidation; ROS	Increased neuro-inflammation, oxidative stress, pro-apoptosis signaling, further insulin resistance White matter atrophy due to fiber and myelin loss
Insulin/IGF availability	Trophic factor withdrawal	Death or impaired function of insulin/IGF dependent neurons and glial cells
Hyperglycemia	Accumulation of advanced glycation end-products	Disrupts removal of Aβ42

**Abbreviations**: GLUT4=glucose transporter 4; IRS= insulin receptor substrate; PI3K= phosphoinositol-3- kinase; GSK-3β = glycogen synthase kinase 3β; GAPDH=glyceraldehyde-3-phosphate dehydrogenase; ROS=reactive oxygen species; RNS=reactive nitrogen species; AβPP= amyloid-β - precursor protein; Aβ 42=amyloid beta peptide-42 amino acids 1-42 cleavage product; IGF=insulin-like growth factor.

**Table 2.** Neuropathologic Processes Contributing to Brain Insulin Resistance in Alzheimer's Disease

Neurodegenerative Disease Process	Mechanism of impairing brain insulin signaling	Consequences in relation to brain insulin signaling
Aβ42 toxicity	Competes with insulin and reduces affinity of insulin binding to its receptor AβPP oligomers desensitize and reduce surface expression of insulin receptors Interferes with PI3K activation of Akt	Disrupts insulin signaling Impairs insulin stimulated neuronal survival and plasticity Increases GSK-3β activation and tau hyperphosphorylation
Microvascular disease	Cerebral hypoperfusion, hypoxic-ischemic injury	Exacerbates insulin resistance;
Oxidative stress	DNA damage, lipid peroxidation, fibrillarization of oligomeric tau and Aβ42	Increases neuro-inflammation and pro-inflammatory cytokine inhibition of insulin signaling Toxic lipids impair signaling through PI3K-Akt
Transition metal ion accumulations	Mitochondrial dysfunction, oxidative stress, tau and AβPP oligomer fibrillarization	Impairs glucose uptake and utilization, inhibits insulin signaling
Hyperphosphorylated-ubiquitinated tau	Increases oxidative stress, promotes neuro-inflammation	Enhances insulin resistance

**Abbreviations**: PI3K= phosphoinositol-3- kinase; GSK-3β = glycogen synthase kinase 3β; AβPP= amyloid-β - precursor protein; Aβ 42=amyloid beta peptide-42 amino acids 1-42 cleavage product

**Table 3.** Therapeutic Targets for Alzheimer's Disease Based on Metabolic Hypothesis

Target	Agent	Mechanism of Action
Glutamate excitotoxicity	NMDA glutamatergic receptor antoginist	Helps restore brain metabolic functions
Cholinergic deficiency	Acetylcholinesterase inhibitor	May stimulate production of trophic factors, e.g IGF-1; protects against glutamate neurotoxicity; activates PI3K-Akt, promoting neuronal survival
Aβ42 accumulation and fibrillarization	Gamma secretase inhibitor drugs (Notch sparing); BACE1 inhibitors to reduce cleavage and production of toxic peptides	Reduces insulin resistance, enhances PI3K-Akt signaling; reduces GSK-3β activity resulting in decreased tau phosphorylation
Tau hyperphosphorylation	GSK-3β and protein phosphatase 2A inhibitors	Reduces oxidative stress, helps restore insulin responsiveness
Insulin deficiency	Insulin therapy-intranasal Incretins, e.g. GLP-1 to stimulate insulin	Maintains survival and function of cells requiring insulin stimulation; supports glucose uptake, brain metabolism and neuronal plasticity; Decreases AβPP burden and tau hyperphosphorylation; Enhances cognition
Hyperglycemia	Antihyperglycemic agents-biguanides	Enhance glucose uptake and insulin receptor sensitivity
Insulin resistance	Insulin sensitizers, e.g. PPAR agonists	Enhance glucose uptake and insulin receptor sensitivity; anti-inflammatory and anti-oxidant properties
Oxidative stress and Neuro-inflammation	Anti-oxidants Radical scavengers Anti-inflammatory agents Transition metal chelators	Help restore insulin sensitivity and glucose utilization Reduce Aβ42 deposition Reduce Aβ42 and tau fibrillarization Reduce cytokine activation-mediated injury Supports microvascular function and cerebral perfusion

**Abbreviations**: BACE1=beta site AβPP cleaving enzyme 1; GLP-1=glucagon-like peptide-1; NMDA= N-methyl-D-aspartate; PPAR= peroxisome proliferator-activated receptor; PI3K= phosphoinositol-3- kinase; GSK-3β = glycogen synthase kinase 3β; AβPP= amyloid-β - precursor protein; Aβ 42=amyloid beta peptide-42 amino acids 1-42 cleavage product; IGF=insulin-like growth factor
